# Chemical Constituents and Bioactivities of *Clinacanthus nutans* Aerial Parts

**DOI:** 10.3390/molecules191220382

**Published:** 2014-12-05

**Authors:** Shu-Fen Tu, Rosa Huang Liu, Yuan-Bin Cheng, Yu-Ming Hsu, Ying-Chi Du, Mohamed El-Shazly, Yang-Chang Wu, Fang-Rong Chang

**Affiliations:** 1 Graduate Institute of Natural Products, College of Pharmacy, Kaohsiung Medical University, Kaohsiung 80708, Taiwan; E-Mails: smallelephant96@yahoo.com.tw (S.-F.T.); jmb@kmu.edu.tw (Y.-B.C.); u98531010@kmu.edu.tw (Y.-M.H.); ycdu0626@gmail.com (Y.-C.D.); elshazly444@googlemail.com (M.E.-S.); 2 School of Nutrition, College of Health Care and Management, Chung Shan Medical University, Taichung 40201, Taiwan; E-Mail: rhl@csmu.edu.tw; 3 Research Center for Natural Product and New Drug, Kaohsiung Medical University, Kaohsiung 80708, Taiwan; 4 Department of Pharmacognosy and Natural Products Chemistry, Faculty of Pharmacy, Ain-Shams University, Cairo 11566, Egypt; 5 School of Pharmacy, College of Pharmacy, China Medical University, Taichung 404, Taiwan; 6 Natural Medicinal Products Research Center, China Medical University Hospital, Taichung 404, Taiwan; 7 Center for Molecular Medicine, China Medical University Hospital, Taichung 404, Taiwan; 8 Cancer Center, Kaohsiung Medical University Hospital, Kaohsiung 80708, Taiwan; 9 Research and Development Center of Chinese Herbal Medicines and New Drugs, College of Pharmacy, Kaohsiung Medical University, Kaohsiung 80708, Taiwan; 10 Department of Marine Biotechnology and Resources, National Sun Yat-sen University, Kaohsiung 80708, Taiwan

**Keywords:** *Clinacanthus nutans*, sulfur-containing compound

## Abstract

Four new sulfur-containing compounds, named clinamides A-C (**1**–**3**), and 2-*cis*-entadamide A (**4**), were isolated together with three known compounds from the bioactive ethanol extract of the aerial parts of *Clinacanthus nutans*. These secondary metabolites possess sulfur atoms and acrylamide functionalities. The structures of the isolated components were established by interpretation of their spectroscopic data, especially 1D and 2D NMR.

## 1. Introduction

**Figure 1:**
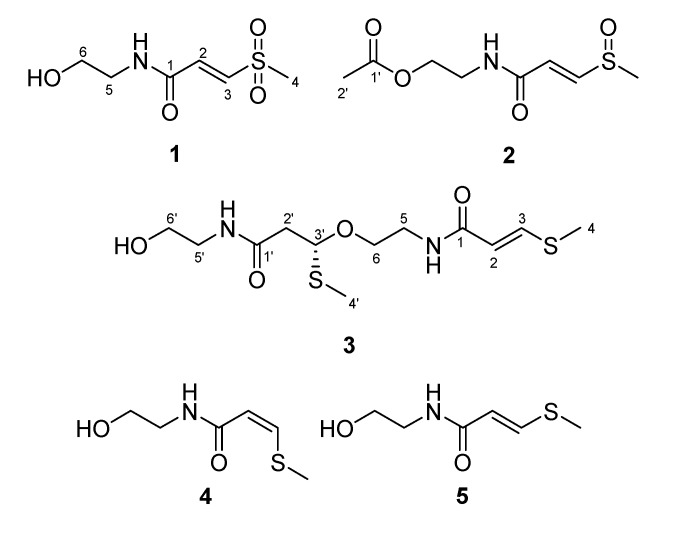
The structures of clinamides A-C (**1**–**3**), 2-*cis*-entadamide A (**4**), and entadamide A (**5**).

The genus *Clinacanthus* (family Acanthaceae) consists of two species. *Clinacanthus nutans* is a small shrub about one meter tall native to tropical Asia [1]. In Malaysia and Thailand, this plant has been claimed to be a useful folk medicine in the treatment of skin rashes, snake bites, herpes simplex virus (HSV), and varicella-zoster virus (VZV) lesions [2]. Actually, extracts from the leaves of *C. nutans* have been reported to possess analgesic, anti-inflammatory, and antiviral activities against VZV and HSV-2 [3]. In previous phytochemical studies, a series of flavonoids, steroids, triterpenoids, cerebrosides, glycoglycero-lipids, glycerides, sulfur-containing glycosides were isolated from this plant [1,2,4]. Among those natural products, glycoglycerolipids and digalactosyl diglycerides exhibited antiviral and anti-HSV activity, respectively [4]. In our bioactive screening, the 80% ethanol extract showed anti-inflammatory, anti-dengue virus, and immune-modulating activity. Those results implied that the active compounds were contained at the 80% ethanol extract. Thus, a phytochemical investigation of *C. nutans* was carried out. Herein, we describe the isolation and structural elucidation of four new sulfur-containing compounds, clinamides A-C (**1**–**3**) and 2-*cis*-entadamide A (**4**) (Figure 1).

## 2. Results and Discussion

Four new compounds **1**–**4** and three known compounds, entadamide A (**5**) [5], entadamide C [6], and *trans*-3-methylsulfinyl-2-propenol [7] were isolated from the ethanolic extract of the aerial parts of *C. nutans*. The known compounds were identified by comparison of their physical and spectroscopic data to those reported in the literature. The structures of the new compounds were established by interpretation of their spectroscopic data, especially 2D NMR.

**Figure 2:**
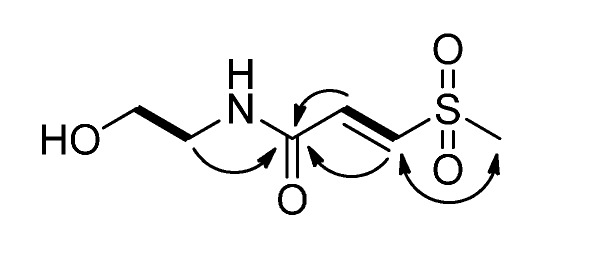
COSY (bold lines), key HMBC (arrows), and NOESY (left right arrow) correlations of **1**.

Clinamide A (**1**) was obtained as a pale yellow oil and was assigned the molecular formula, C_6_H_11_NO_4_S, as deduced from HRESIMS (*m*/*z* 216.0308 [M+Na]^+^). The IR spectrum showed strong absorption bands at 3380 cm^−1^ (OH), 1668 cm^−1^ (NH–C=O), 1555 cm^−1^ (C=C), and 1302 and 1135 cm^−1^ (O=S=O). The ^1^H-NMR data of **1** (Table 1) exhibited one methylsulfonyl signal [δ_H_ 3.08 (s)] [7], two methylene signals [δ_H_ 3.41 (t, *J* = 5.7 Hz) and 3.65 (t, *J* = 5.7 Hz)], and a *trans*-disubstituted double bond [δ_H_ 7.01 (d, *J* = 15.0 Hz)] and 7.43 (d, *J* = 15.0 Hz)]. The ^13^C-NMR (Table 2), DEPT and HSQC spectra of **1** showed six carbon signals, consisting of one methylsulfonyl group (δ_C_ 42.4), one oxymethylene group (δ_C_ 61.2), one methylene group (δ_C_ 43.4), one disubstituted olefin group (δ_C_ 136.3 and 140.1), and one amide carbonyl group (δ_C_ 164.6). These signature characteristics were used to identify **1** as an entadamide-type compound. The COSY spectrum of **1** (Figure 2) exhibited two proton spin systems of H-2 (δ_H_ 7.01)/H-3 (δ_H_ 7.43) and H-5 (δ_H_ 3.41)/H-6 (δ_H_ 3.65). In the HMBC spectrum, the H-2, H-3 and H-5 showed correlations to C-1 (δ_C_ 164.6), which indicated the presence of the double bond, the amide group and two methylene groups were connected. In addition, the key NOESY correlation between H-4 (δ_H_ 3.08) and H-3 indicated the methylsulfonyl group at C-3. These NMR data of **1** were closely similar to dambullin [8], except for the absence of an aromatic moiety. On the basis of the above discussion, the configuration of **1** was established as shown.

**Table 1:** ^1^H-NMR spectroscopic data of compounds **1**–**4** (**1** and **2** in CD_3_OD, **3** and **4** in CDCl_3_, 400 MHz).

NO.	1	2	3	4
2	7.01 (d, 15.0)	6.68 (d, 15.0)	5.78 (d, 14.8)	5.80 (d, 10.0)
3	7.43 (d, 15.0)	7.63 (d, 15.0)	7.62 (d, 14.8)	6.83 (d, 10.0)
4	3.08 (s)	2.76 (s)	2.33 (s)	2.35 (s)
5	3.41 (t, 5.7)	3.54 (t, 5.4)	3.65 (m)	3.46 (t, 5.0)
			3.38 (m)	
6	3.65 (t, 5.7)	4.17 (t, 5.4)	3.88 (m)	3.73 (t, 5.0)
			3.52 (m)	
2'		2.05 (s)	2.80 (dd, 15.0, 9.8)	
			2.70 (dd, 15.0, 3.2)	
3'			4.87 (dd, 9.8, 3.2)	
4'			2.06 (s)	
5'			3.52 (m)	
			3.38 (m)	
6'			3.74 (m)	

**Table 2:** ^13^C-NMR spectroscopic data of compounds **1**–**4** (**1** and **2** in CD_3_OD, **3** and **4** in CDCl_3_, 100 MHz).

NO.	1	2	3	4
1	164.6 (s)	165.2 (s)	165.1 (s)	167.4 (s)
2	136.3 (d)	129.0 (d)	115.9 (d)	114.9 (d)
3	140.1 (d)	147.9 (d)	142.6 (d)	147.8 (d)
4	42.4 (q)	39.9 (q)	14.5 (q)	19.4 (q)
5	43.4 (t)	39.8 (t)	38.9 (t)	42.3 (t)
6	61.2 (t)	63.8 (t)	67.5 (t)	62.4 (t)
1'		172.7 (s)	171.0 (s)	
2'		20.7 (q)	43.3 (t)	
3'			81.4 (d)	
4'			10.2 (q)	
5'			42.2 (t)	
6'			62.1 (t)	

**Figure 3:**
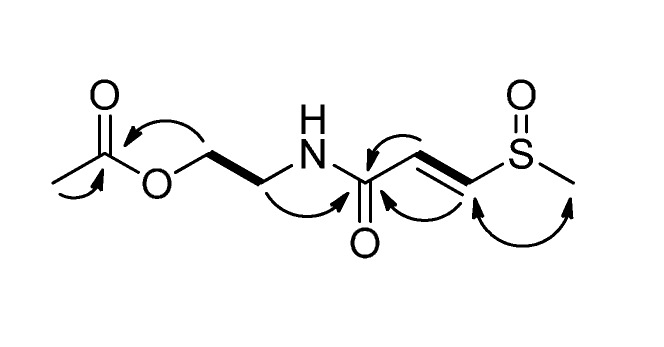
COSY (bold lines), key HMBC (arrows), and NOESY (left right arrow) correlations of **2**.

Clinamide B (**2**) was assigned a molecular formula of C_8_H_13_NO_4_S by HRESIMS (*m*/*z* 242.0461 [M+Na]^+^). The UV spectrum exhibited absorption maximum at 247 nm. The IR spectrum showed bands at 1732 cm^−1^ (C=O), 1,660 cm^−1^ (NH–C=O), and 1,031 cm^−1^ (S=O). In the ^1^H-NMR spectrum (Table 1), a pair of *trans*-disubstituted olefinic protons [δ_H_ 6.68 (d, *J* = 15.0 Hz) and 7.63 (d, *J* = 15.0 Hz)] were found as similar to the case of **1**. Besides, the proton spectrum showed a methylsulfinyl group [δ_H_ 2.76 (s)], a methyl group [δ_H_ 2.05 (s)], and two methylene groups [δ_H_ 3.54 (t, *J* = 5.4 Hz) and 4.17 (t, *J* = 5.4 Hz)]. The ^13^C-NMR (Table 2) spectrum revealed signals for an amide group as well as **1**. In addition, the ^13^C-NMR and DEPT spectra exhibited one amide carbon group (δ_C_ 172.7), two methine groups (δ_C_ 129.0 and 147.9), two methylene groups (δ_C_ 39.8 and 63.8), and two methyl groups (δ_C_ 20.7 and 39.9). These signals were similar to entadamide C, suggesting that they possess the same skeleton. Compound **2** was found to have an additional acetyl group, when compared to entadamide C. This acetoxy group was assigned at C-6 by the HMBC correlations from both H-2' (δ_H_ 2.05) and H-6 (δ_H_ 4.17) to C-1' (δ_C_ 172.7) (Figure 3). Therefore, structure **2** was assigned to clinamide B.

**Figure 4:**
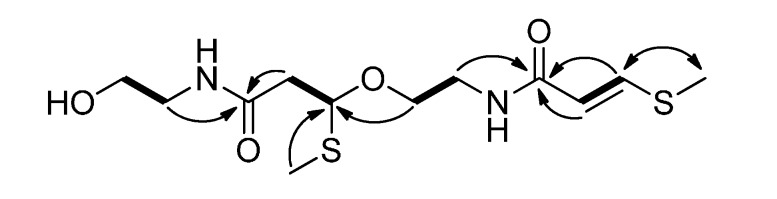
COSY (bold lines), key HMBC (arrows), and NOESY (left right arrow) correlations of **3**.

Clinamide C (**3**) was isolated as pale yellow oil, and exhibited the molecular formula C_12_H_22_N_2_O_4_S_2_ by HRESIMS. The UV spectrum exhibited absorption maxima at 228 nm and 270 nm. The IR spectrum showed absorption bands at 3,296 cm^−1^ (OH), 1,644 cm^−1^ (NH–C=O), and 1,582 cm^−1^ (C=C). The ^1^H-NMR data (Table 1) showed two methylthio signals [δ_H_ 2.06 (s) and 2.33 (s)], and a *trans*-disubstituted double bond [δ_H_ 5.78 (d, *J* = 14.8 Hz) and 7.62 (d, *J* = 14.8 Hz)]. The ^13^C-NMR (Table 2), DEPT and HSQC spectra exhibited signals for 12 carbons, including two methylthio groups (δ_C_ 10.2 and 14.5), five methylene groups (δ_C_ 38.9, 42.2, 43.3, 62.1, and 67.5), one disubstituted olefin (δ_C_ 115.9 and 142.6), and two amide carbonyl groups (δ_C_ 165.1 and 171.0). The partial NMR spectroscopic data of **3** were close to those of entadamide A (**5**) [5]. The COSY spectrum (Figure 4) of **3** indicated the presence of fragments of H-2/H-3, H-5/H-6, H-2'/H-3', and H-5'/H-6'. The connectivity between two amide groups and above fragments were linked by HMBC correlations of H-2/C-1, H-3/C-1, H-5/C-1, H-4/C-3, H-2'/C-1', H-5'/C-1', H-3'/C-4', H-4'/C-3', and H-6/C-3'. The optical rotation value of **3** (−10.7) was opposite to the synthetic compound (*S*)-4-methoxy-4-(phenylthio)-2-butanone (+53.9). Thus, the relative configuration of C-3' was proposed to be R* on the basis of its optical rotation result. According to the above interpretation, the structure of **3** was established as clinamide C.

The HRESIMS indicated a molecular formula C_6_H_11_NO_2_S for compound **4**, identical to that of **5**. The ^1^H and ^13^C-NMR data of **4** were similar to those of **5**, suggesting that they should be geometric isomers. The ^1^H-NMR spectrum of **4** exhibited the signals of a *cis-*double bond at [δ_H_ 5.80 (d, *J* = 10.0 Hz) and 6.83 (d, *J* = 10.0 Hz)]; however, a *trans*-one at [δ_H_ 5.85 (d, *J* = 14.8 Hz) and 7.56 (d, *J* = 14.8 Hz)] were found in that of **5**. Thus, the structure of 2-*cis*-entadamide A (**4**) was determined as shown.

The results of the biological activity were discussed in the Conclusion section and the bioactivity tables were put in supporting information. 

## 3. Experimental Section

### 3.1. General Procedures

Optical rotations were measured with a JASCO DIP-370 digital polarimeter. The IR spectra were measured on a Mattson Genesis II spectrometer. ^1^H and ^13^C-NMR spectra were recorded on Varian Unity Inova-600, Varian Unity Plus-400, or Varian Gemini 2000-200 NMR spectrometers. Chemical shifts are reported in parts per million (δ), and coupling constants (*J*) are expressed in Hertz. LRESIMS were measured on a VG Biotech Quattro 5022 mass spectrometer. HRESIMS were measured on a Bruker Daltonics APEX II mass spectrometer. Silica gel 60 (Merck, 230–400 mesh) and Sephadex LH-20 were used for column chromatography, while TLC analysis was carried out on silica gel GF_254_ pre-coated plates with detection using 50% H_2_SO_4_ followed by heating on a hot plate. HPLC was performed with Hitachi L-7100 series HPLC with a Bischoff RI detector and Shimadzu LC-10AT series HPLC with a SPD-10A UV-vis detector or a photodiode array detector. ODS (Hypersil^®^, 250 × 10 mm) columns were applied for HPLC separation.

### 3.2. Plant Material

The aerial parts of *Clinacanthus nutans* were collected in Taichung, Taiwan, in November 2010, and identified by Ming-Hong Yen. The samples were deposited at the Graduate Institute of Natural Products, Kaohsiung, Taiwan.

### 3.3. Extraction and Isolation

The aerial parts of *C. nutans* (2.3 kg) were collected freshly, cut into small pieces (*ca.* 0.5–1.0 cm thickness), and air-dried for three days. After drying, the materials were soaked and extracted with ethanol (10 L) at room temperature for three times, and then concentrated under reduced pressure to afford a crude extract (283.0 g). This crude extract was partitioned between EtOAc and H_2_O (1:1) to obtain an EtOAc-soluble layer. After evaporating the organic solvent, the EtOAc residue (51.1 g) was partitioned between *n*-hexane–EtOH–H_2_O (5:4:1) to afford an 80% ethanol extract. The 80% ethanol extract (14.6 g) was subjected to passage over a silica gel column using gradient elution of CH_2_Cl_2_–MeOH (1:0 to 5:1) to furnish fractions 1–20. Fraction 13 (618.5 mg) was separated on a Sephadex LH-20 column (5 × 55 cm) eluted with EtOAc–CH_2_Cl_2_–MeOH (1:1:6) to afford fraction 13-5 (298.0 mg), which was further chromatographied on a silica gel column (3 × 25 cm; CH_2_Cl_2_–MeOH, 20:1) to furnish subfractions 13-5-1 to 13-5-10. Compound **6** (16.0 mg) was obtained by separation of subfraction 13-5-8.9 (25.5 mg) on preparative TLC eluting with CH_2_Cl_2_–MeOH (6:1). Subfraction 13-5-4 (149.6 mg) was chromatographed by preparative TLC using CH_2_Cl_2_–MeOH (8:1) for elution, and further purified by reverse-phase HPLC (270 nm; Hypersil^®^, 250 × 10 mm) with MeOH–H_2_O (35:65) to yield **3** (3.8 mg; flow rate: 2 mL/min; *R_t_* 11.5 min). Then the subfraction was eluted with MeOH–H_2_O (40:60) to yield **4** (6.8 mg; flow rate: 2 mL/min; *R_t_* 8 min) as well as **5** (99.9 mg; flow rate: 2 mL/min; *R_t_* 10 min). Fraction 15 (1035.1 mg) was purified on a Sephadex LH-20 column (5 × 55 cm) using EtOAc–CH_2_Cl_2_–MeOH (1:1:6) as eluting solvent to afford nine subfractions. Subfraction 15-5 (296.0 mg) was applied to a silica gel column (3 × 25 cm) eluted with CH_2_Cl_2_–MeOH (30:1) to give ten subfractions. Compound **2** was obtained by separation of subfraction 15-5-4 (40.1 mg) on preparative TLC eluting with CH_2_Cl_2_–MeOH (10:1) and further purification by reverse-phase HPLC (244 nm; Hypersil^®^, 250 × 10 mm) with MeOH–H_2_O (43:57) (0.8 mg; flow rate: 2 mL/min; *R_t_* 7.5 min). Subfraction 15-5-7 (29.1 mg) was subjected to preparative TLC with CH_2_Cl_2_–MeOH (15:1) and further purified by reverse-phase HPLC (245 nm; Hypersil^®^, 250 × 10 mm) with MeOH–H_2_O (25:75) to yield **7** (14.2 mg; flow rate: 2 mL/min; *R_t_* 9 min) and with MeOH–H_2_O (15:85) to yield **1** (2.2 mg; flow rate: 2 mL/min; *R_t_* 13 min).

*Clinamide A* (**1**): Pale yellow oil; IR (neat): *v*_max_ 3380, 2927, 1668, 1555, 1302, 1135 cm^−1^; ^1^H-NMR (CD_3_OD, 400 MHz) and ^13^C-NMR (CD_3_OD, 100 MHz) spectroscopic data, see Table 1 and Table 2, respectively; HRESIMS: *m*/*z* 216.0308 [M+Na]^+^ (calcd. for C_6_H_11_NO_4_SNa, 216.0306).

*Clinamide B* (**2**): White amorphous powder; IR (neat): *v*_max_ 2918, 1732, 1031 cm^−1^; ^1^H-NMR (CD_3_OD, 400 MHz) and ^13^C-NMR (CD_3_OD, 100 MHz) spectroscopic data, see Table 1 and Table 2, respectively; HRESIMS: *m*/*z* 242.0461 [M+Na]^+^ (calcd. for C_8_H_13_NO_4_SNa, 242.0463).

*Clinamide C* (**3**): Pale yellow oil; ${\left[\text{α}\right]}_{\text{D}}^{25}$ −10.7 (*c* 1.0, MeOH); IR (neat): *v*_max_ 3296, 2924, 1644, 1582 cm^−1^; ^1^H-NMR (CDCl_3_, 400 MHz) and ^13^C-NMR (CDCl_3_, 100 MHz) spectroscopic data, see Table 1 and Table 2, respectively; HRESIMS: *m*/*z* 345.0903 [M+Na]^+^ (calcd. for C_12_H_22_N_2_O_4_S_2_Na, 345.0905).

*2-cis-Entadamide A* (**4**): Pale yellow oil; IR (neat): *v*_max_ 3306, 2924, 1644, 1583 cm^−1^; ^1^H-NMR (CDCl_3_, 400 MHz) and ^13^C-NMR (CDCl_3_, 100 MHz) spectroscopic data, see Table 1 and Table 2, respectively; HRESIMS: *m*/*z* 184.0409 [M+Na]^+^ (calcd. for C_6_H_11_NO_2_SNa, 184.0408).

### 3.4. Anti-Inflammatory Assay

Human neutrophils were collected by means of Ficoll centrifugation and dextran sedimentation. Assessments of superoxide anion generation and elastase release were carried out according to known procedures [9,10]. In brief, superoxide anion production was assayed by monitoring the superoxide dismutase-inhibitable reduction of ferricytochrome *c*. Elastase release experiments were executed using MeO-Suc-Ala-Ala-Pro-Valp-nitroanilide as the elastase substrate. In the *in vitro* anti-inflammatory bioassay, the inhibitory effects on the generation of superoxide anion and the release of elastase by activated neutrophils were used as indicators. For significant activity of pure compounds, an inhibition rate ≥50% is required.

### 3.5. Anti-Dengue Virus Assay

Naïve Huh-7 cells were obtained from American type culture collection (ATCC) and cultured in Dulbecco’s modified Eagle’s medium (DMEM) containing 10% fetal bovine serum, 1% non-essential amino acids, and 1% antibiotic-antimycotic in a 5% CO_2_ in air atmosphere at 37 °C. Huh-7 cells were seeded at 24 wells plate at density 5 × 10^4^ cells/well for 12–16 h and then infected with DENV-2, strain 16681 at a multiplicity of infection (MOI) of 0.2 for 2 h at 37 °C. Cells were washed with PBS and then re-fed with DMEM-10% FBS medium containing test compounds. Cells were harvested at 72 h post-infection on a western blot assay with anti-DENV NS2B (1:2000; Abcam, Cambridge, MA, USA) or anti-GAPDH antibody (1:10000; GeneTex, Irvine, CA, USA), a loading control. The signal was detected using an ECL detection kit (PerkinElmer, Shelton, CT, USA). Ribavirin served as positive control [11].

### 3.6. Immune-Modulating Assay

To examine the inhibitory effect of *Lactobacillus casei* on IgE production, splenocyte obtained from ovalbumin (OVA)-primed BALB/c mice were re-stimulated *in vitro* with the same antigen in the presence of heat-killed *L. casei*. The effect of this bacterium on helper (Th) phenotype development was also examined with native T cells from T cell receptor-transgenic mice [12].

## 4. Conclusions

The plant material and extracts of *C. nutans* have now become popular commercial products in Thailand and Malaysia, even available through Internet markets worldwide, and dubitatively used in anticancer and anti-cardiovascular diseases, making the active components from this plant an important issue to be understood. Our current study enhances the bioactivity and chemical diversity of this plant material. In bioactive screening, the 80% ethanol extract showed anti-inflammatory, anti-dengue virus and immune-modulating activity. For the study of anti-inflammatory activity, the extract at 10 μg/mL had the strongest elastase release inhibitory effect at 68.33%. Moreover, this extract displayed moderate anti-dengue virus activity in the IC_50_ 31.04 μg/mL. In an immune-modulating experiment, using 0.1 μg/mL of 80% ethanol extract led to up-regulation of IFN-γ. On the other hand, using high concentration of 80% ethanol extract (100 μg/mL) led to down-regulation of IFN-γ exhibiting immune-modulating activity. Unfortunately, the new compounds isolated so far did not showed any targeted activity.

## Supplementary Materials

Supplementary materials can be found at http://www.mdpi.com/1420-3049/19/12/20382/s1.
