# Supplementary material for: Chemical Constituents and Bioactivities of Clinacanthus nutans Aerial Parts
Source: Molecules. 2014 Dec 5;19(12):20382–90. doi: 10.3390/molecules191220382 (PMC6271080; doi:10.3390/molecules191220382)

Supplementary Information

Contents

**Figure S1.** ^{1}H-NMR Spectrum of **1** in CD_3OD	S2
**Figure S2.** ^{13}C-NMR Spectrum of **1** in CD_3OD	S3
**Figure S3.** DEPT Spectrum of **1** in CD_3OD	S4
**Figure S4.** COSY Spectrum of **1** in CD_3OD	S5
**Figure S5.** HMQC Spectrum of **1** in CD_3OD	S6
**Figure S6.** HMBC Spectrum of **1** in CD_3OD	S7
**Figure S7.** NOESY Spectrum of **1** in CD_3OD	S8
**Figure S8.** ^{1}H-NMR Spectrum of **2** in CD_3OD	S9
**Figure S9.** ^{13}C-NMR Spectrum of **2** in CD_3OD	S10
**Figure S10.** DEPT Spectrum of **2** in CD_3OD	S11
**Figure S11.** COSY Spectrum of **2** in CD_3OD	S12
**Figure S12.** HMQC Spectrum of **2** in CD_3OD	S13
**Figure S13.** HMBC Spectrum of **2** in CD_3OD	S14
**Figure S14.** NOESY Spectrum of **2** in CD_3OD	S15
**Figure S15.** ^{1}H-NMR Spectrum of **3** in $CDCl_3$	S16
**Figure S16.** ^{13}C-NMR Spectrum of **3** in $CDCl_3$	S17
**Figure S17.** DEPT Spectrum of **3** in $CDCl_3$	S18
**Figure S18.** COSY Spectrum of **3** in $CDCl_3$	S19
**Figure S19.** HMQC Spectrum of **3** in $CDCl_3$	S20
**Figure S20.** HMBC Spectrum of **3** in $CDCl_3$	S21
**Figure S21.** NOESY Spectrum of **3** in $CDCl_3$	S22
**Figure S22.** ^{1}H-NMR Spectrum of **4** in $CDCl_3$	S23
**Figure S23.** ^{13}C-NMR Spectrum of **4** in $CDCl_3$	S24
**Table S1.** Anti-inflammatory effects of ethanol extraction of *C. nutans*	S25
**Figure S24.** Anti-dengue virus 2 result of ethanol extraction of *C. nutans*	S25
**Figure S25.** Immune-modulating result of 80% EtOH layer	S25

Figure S1. ^{1}H-NMR Spectrum of **1** in CD_3OD.

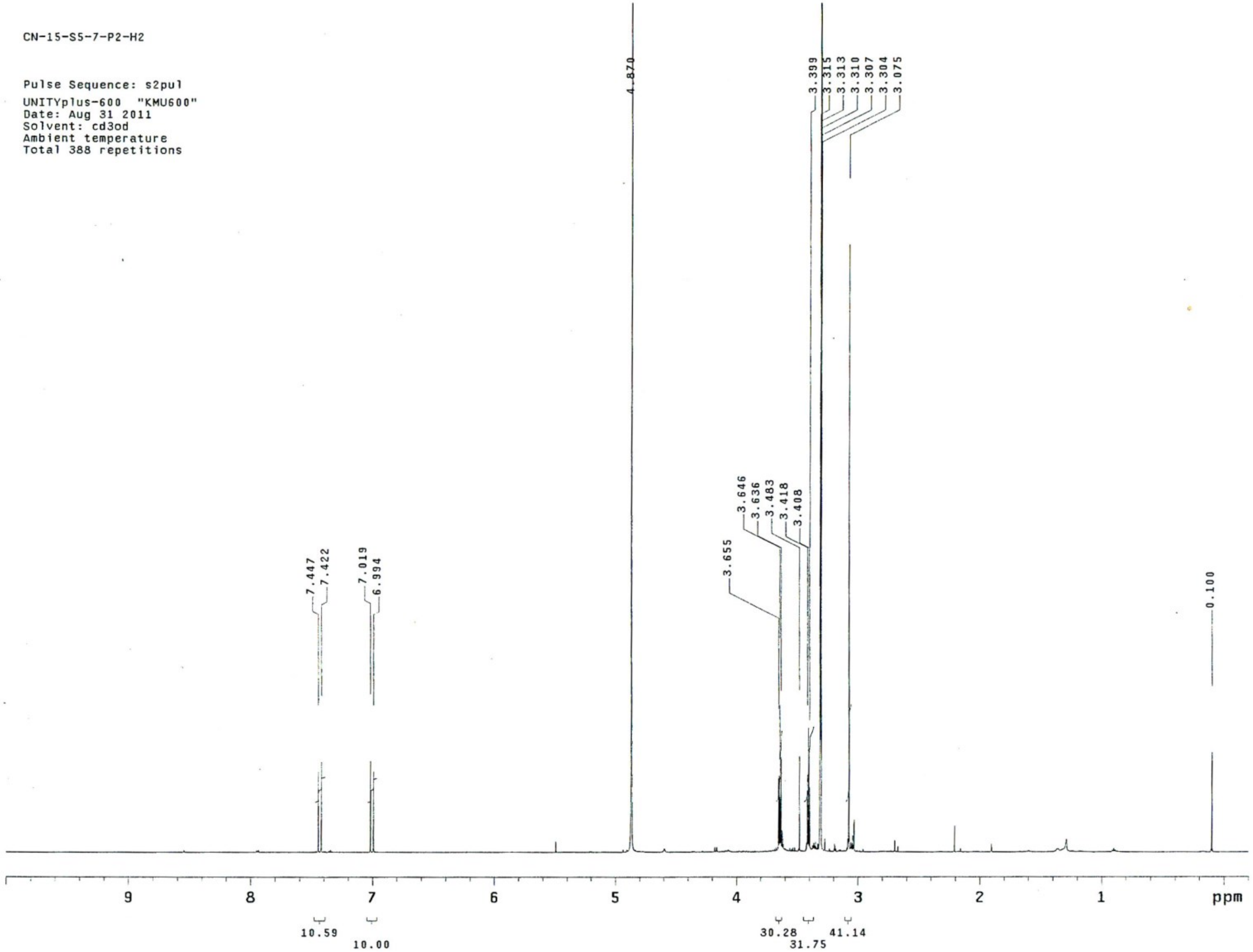

Figure S2. ^{13}C-NMR Spectrum of **1** in CD_3OD.

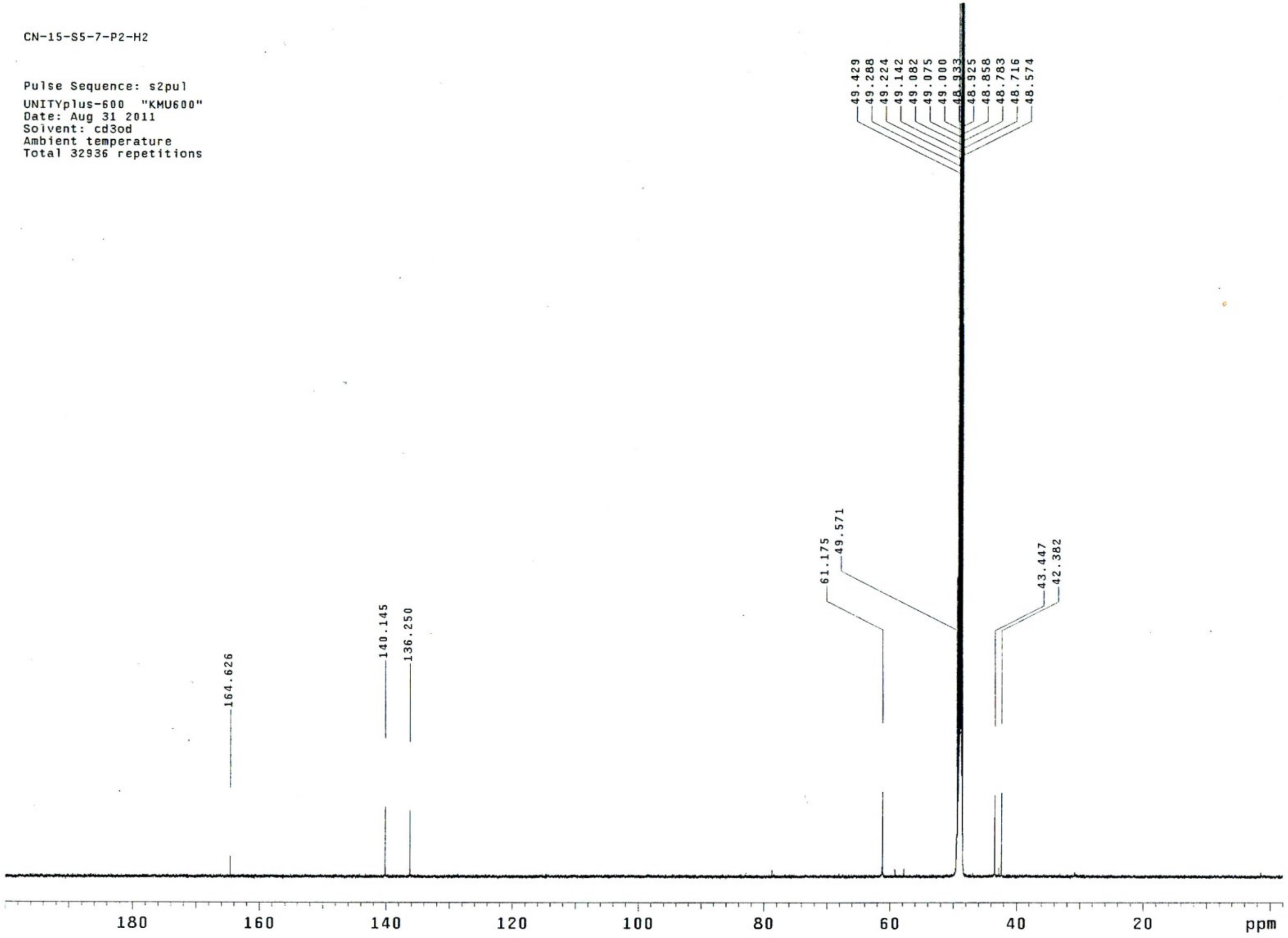

Figure S3. DEPT Spectrum of **1** in CD_3OD.

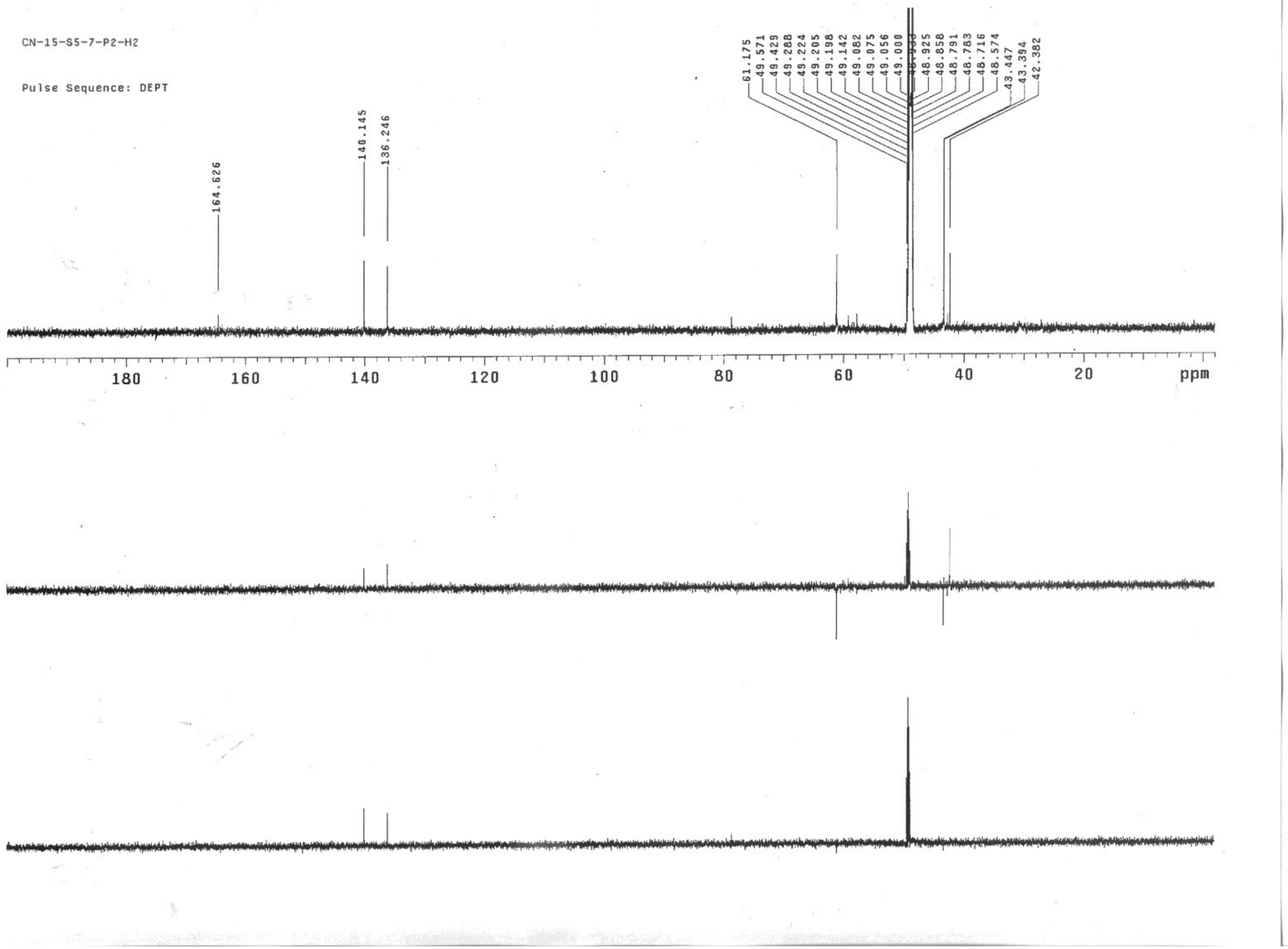

Figure S4. COSY Spectrum of **1** in CD_3OD.

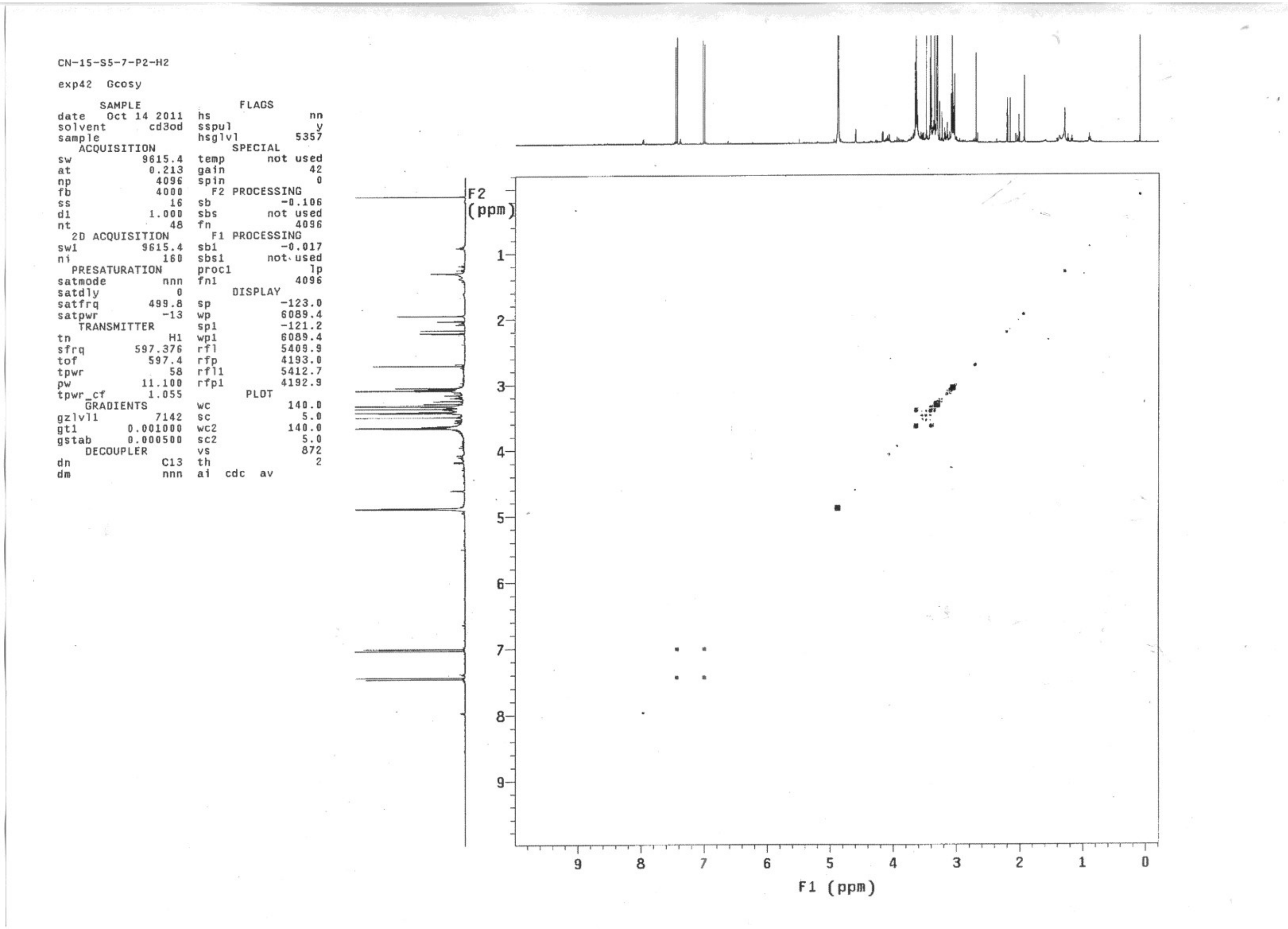

Figure S5. HMQC Spectrum of **1** in CD_3OD.

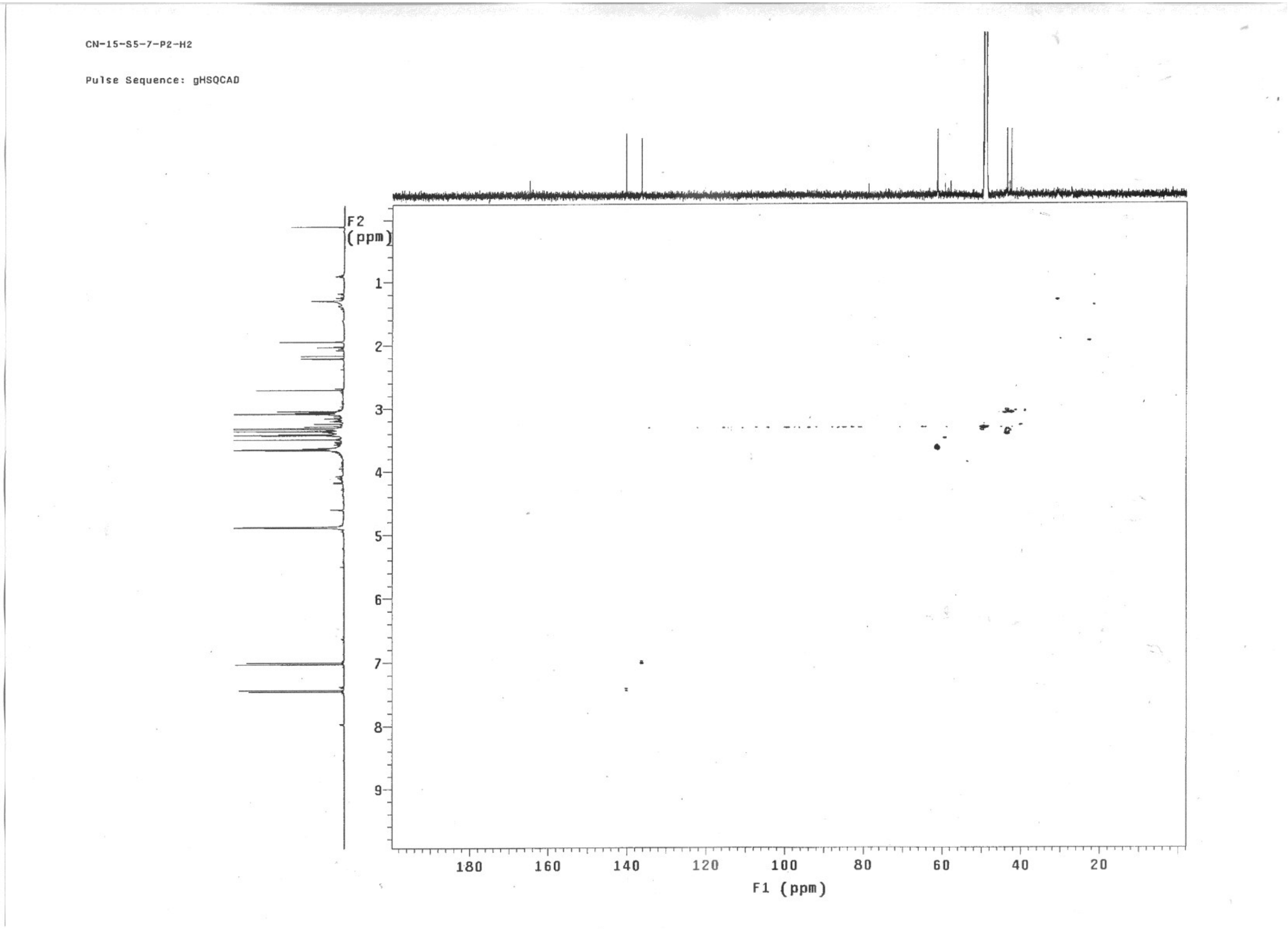

Figure S6. HMBC Spectrum of **1** in CD_3OD.

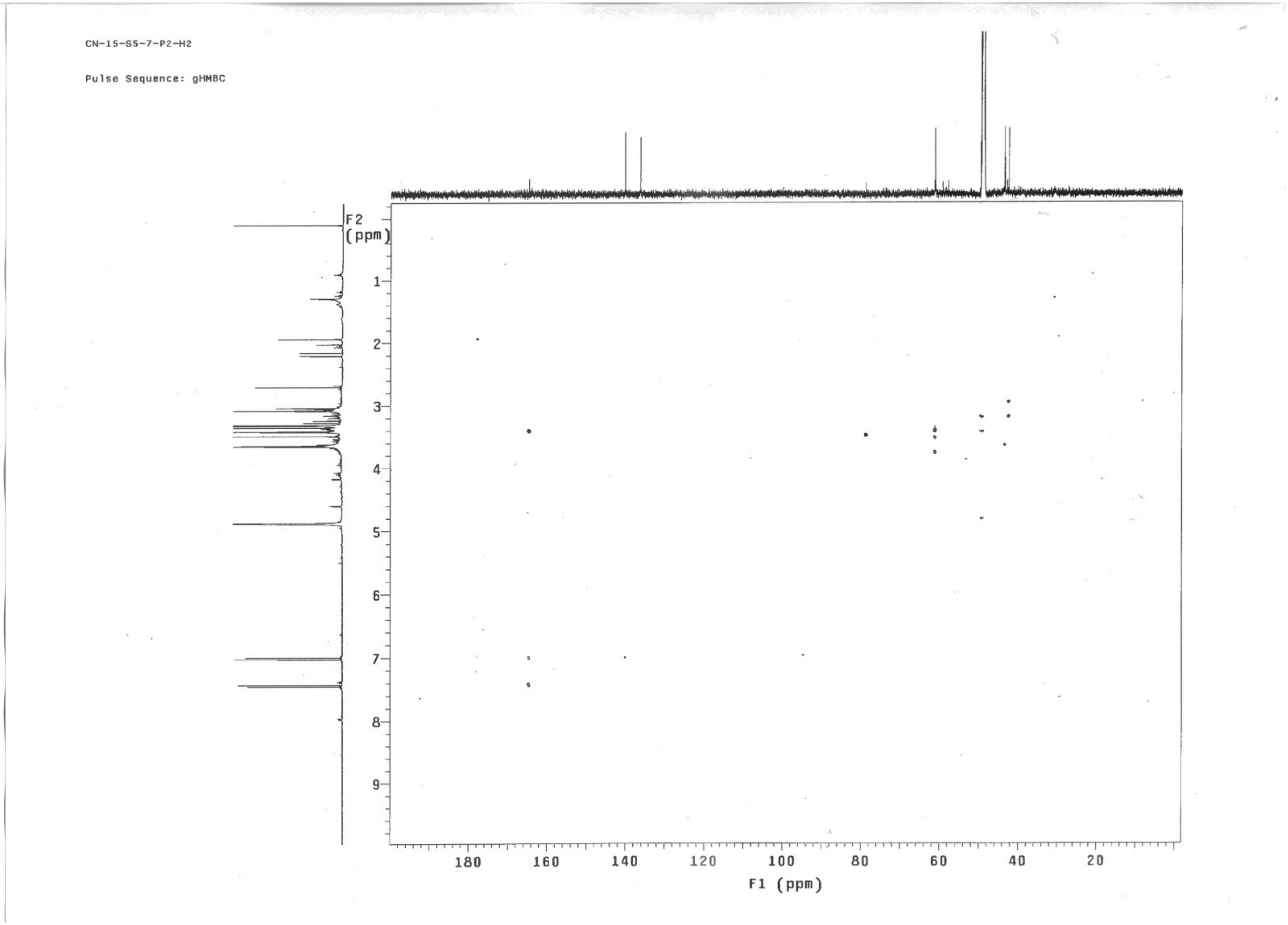

Figure S7. NOESY Spectrum of **1** in CD_3OD.

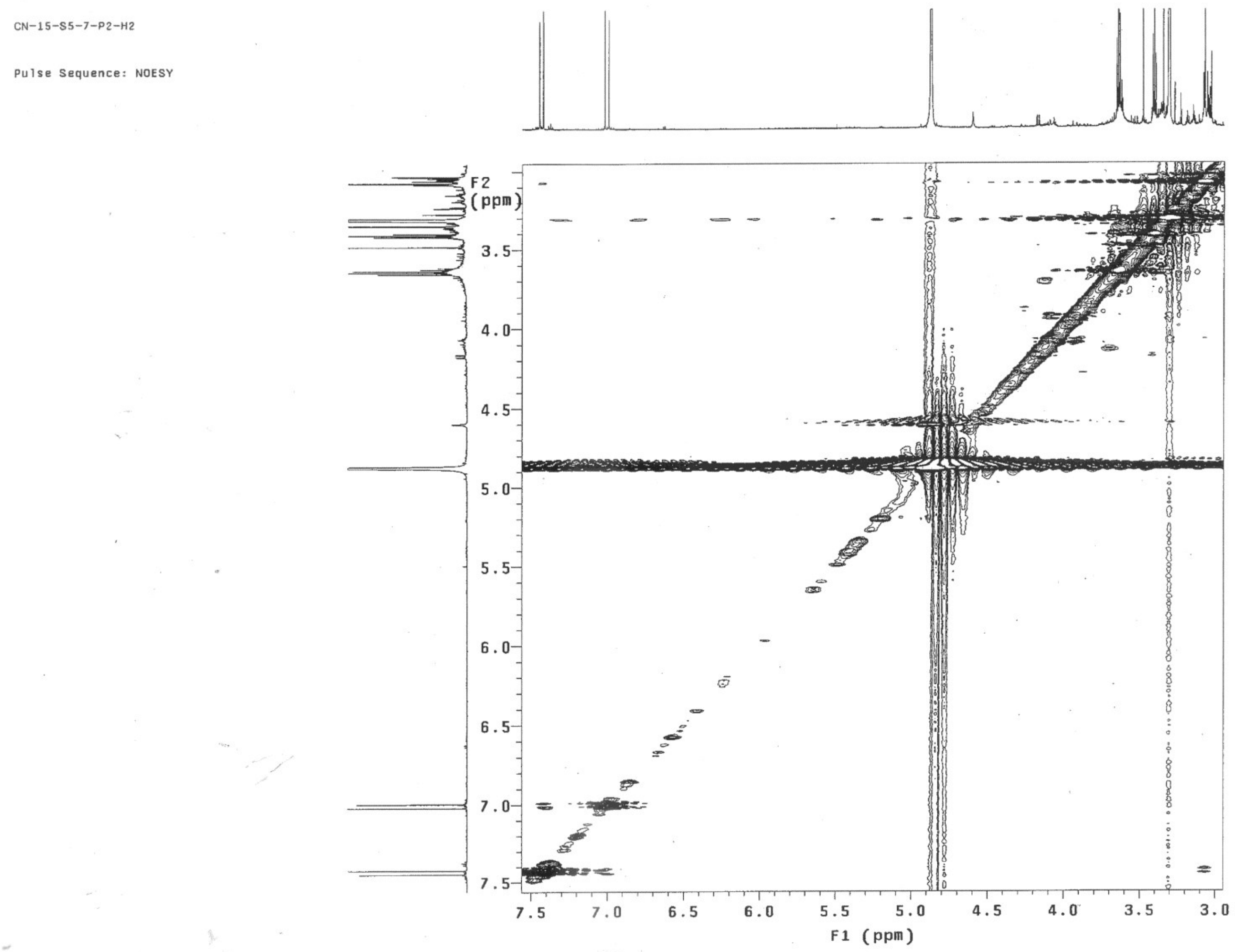

Figure S8. ^{1}H-NMR Spectrum of **2** in CD_3OD.

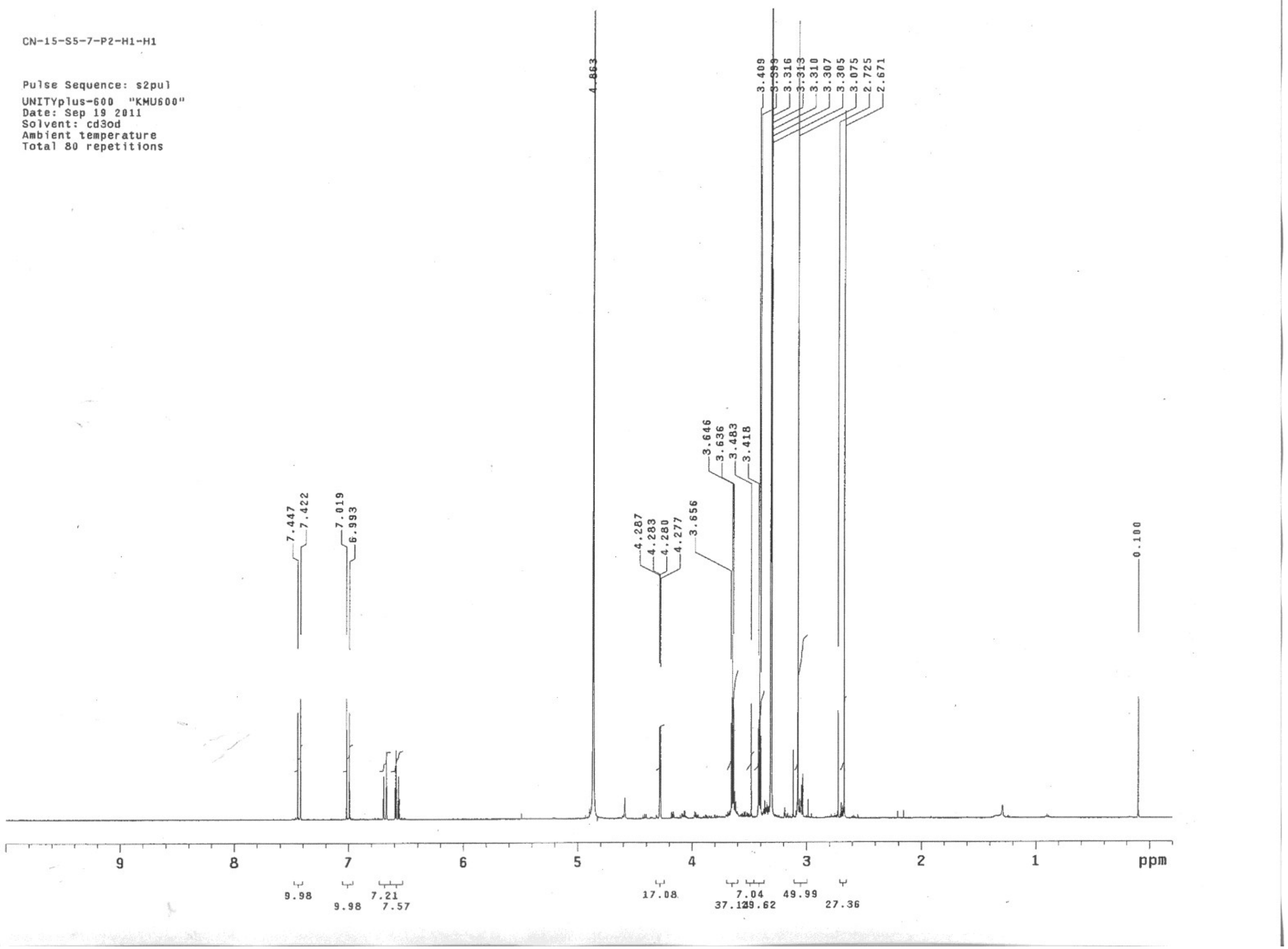

Figure S9. ^{13}C-NMR Spectrum of **2** in CD_3OD.

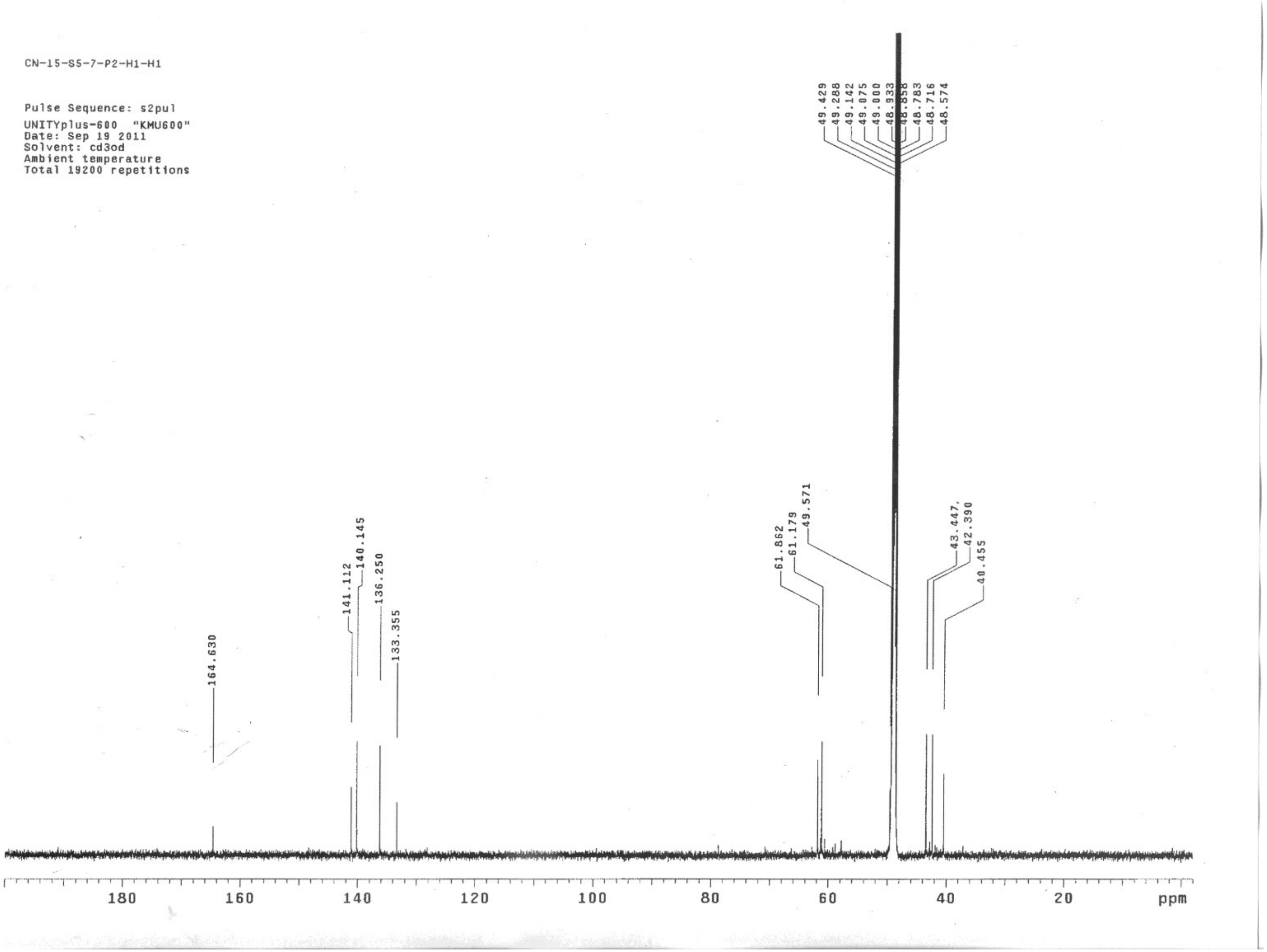

Figure S10. DEPT Spectrum of **2** in CD_3OD.

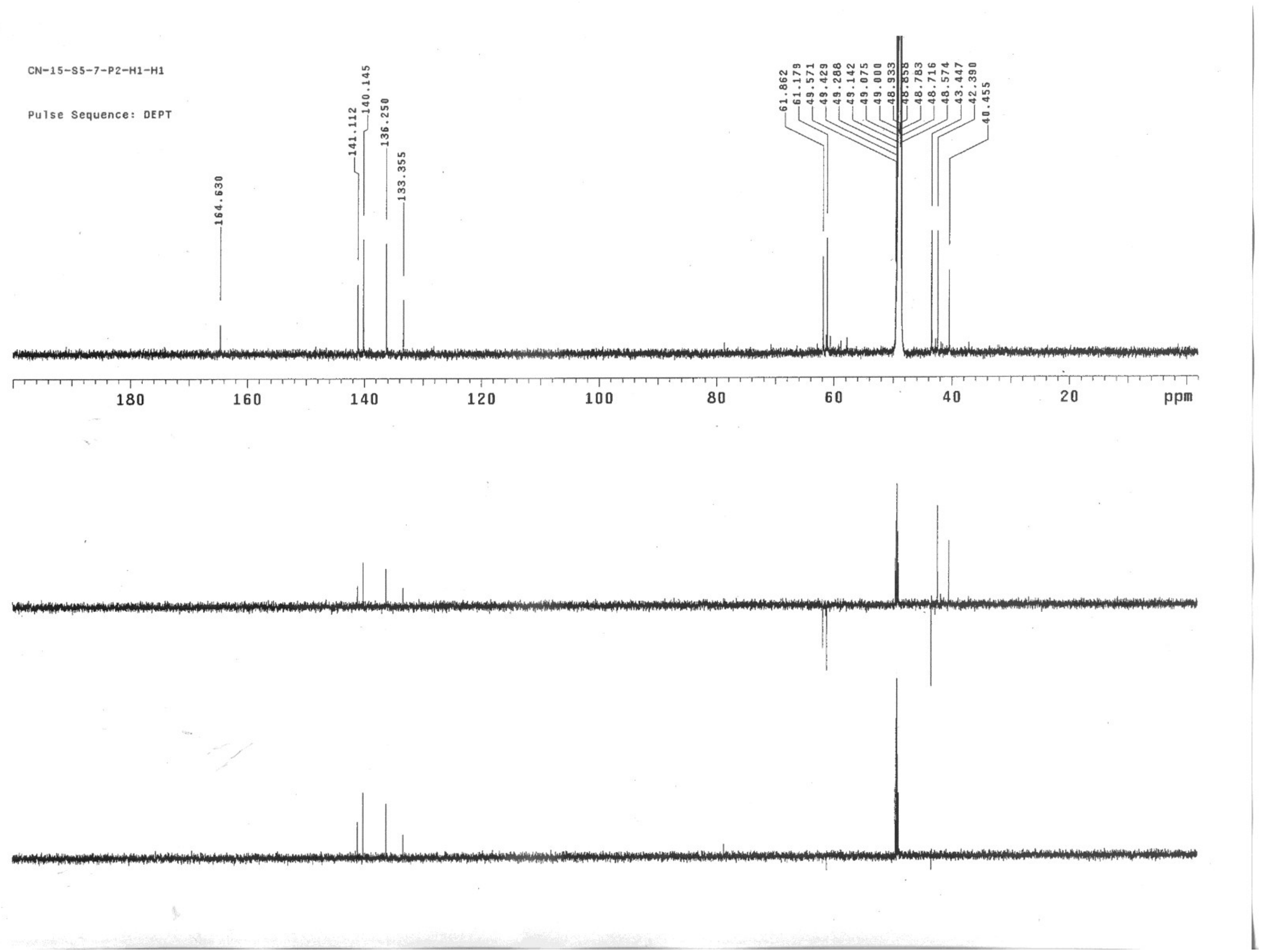

Figure S11. COSY Spectrum of **2** in CD_3OD.

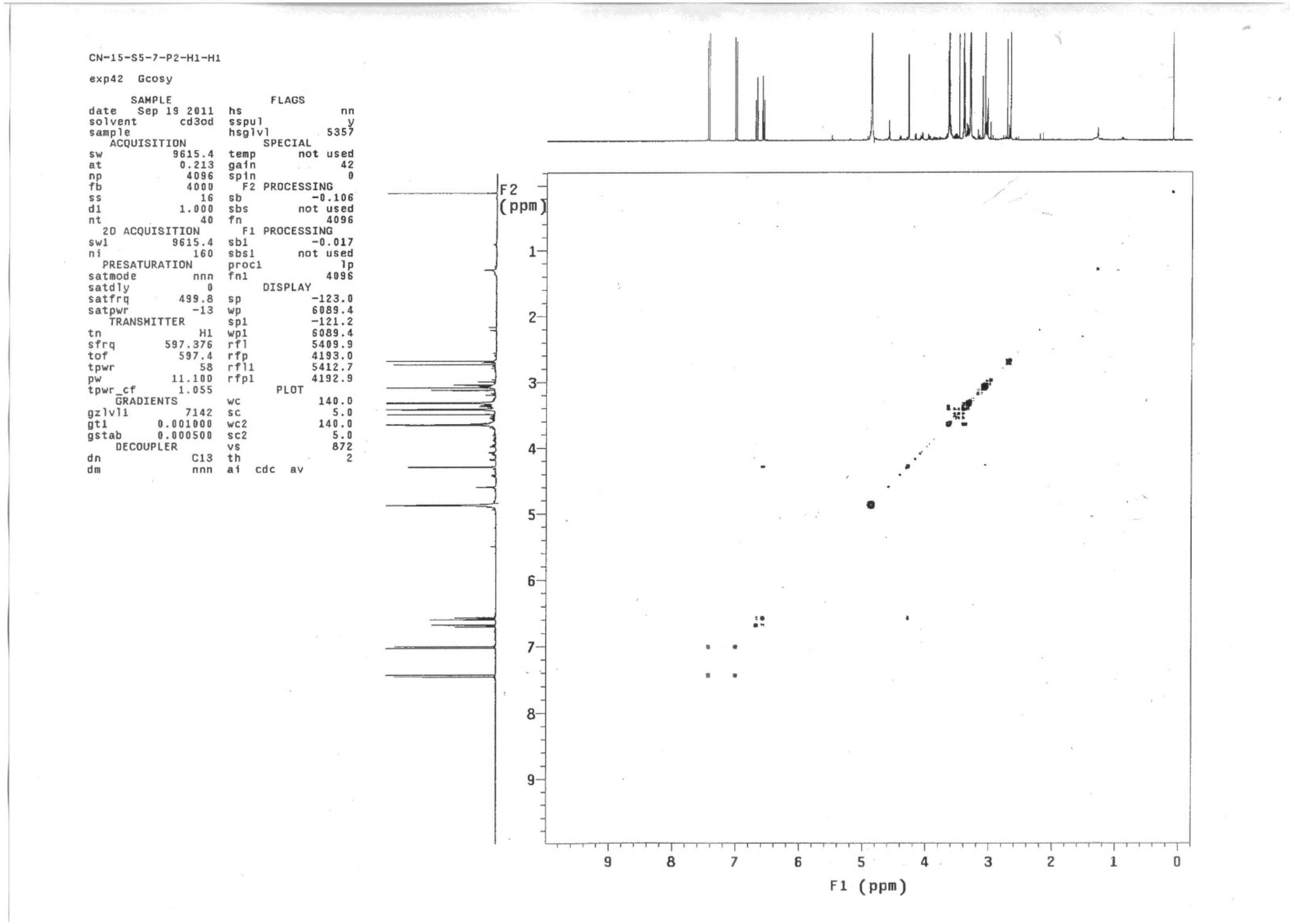

Figure S12. HMQC Spectrum of **2** in CD_3OD.

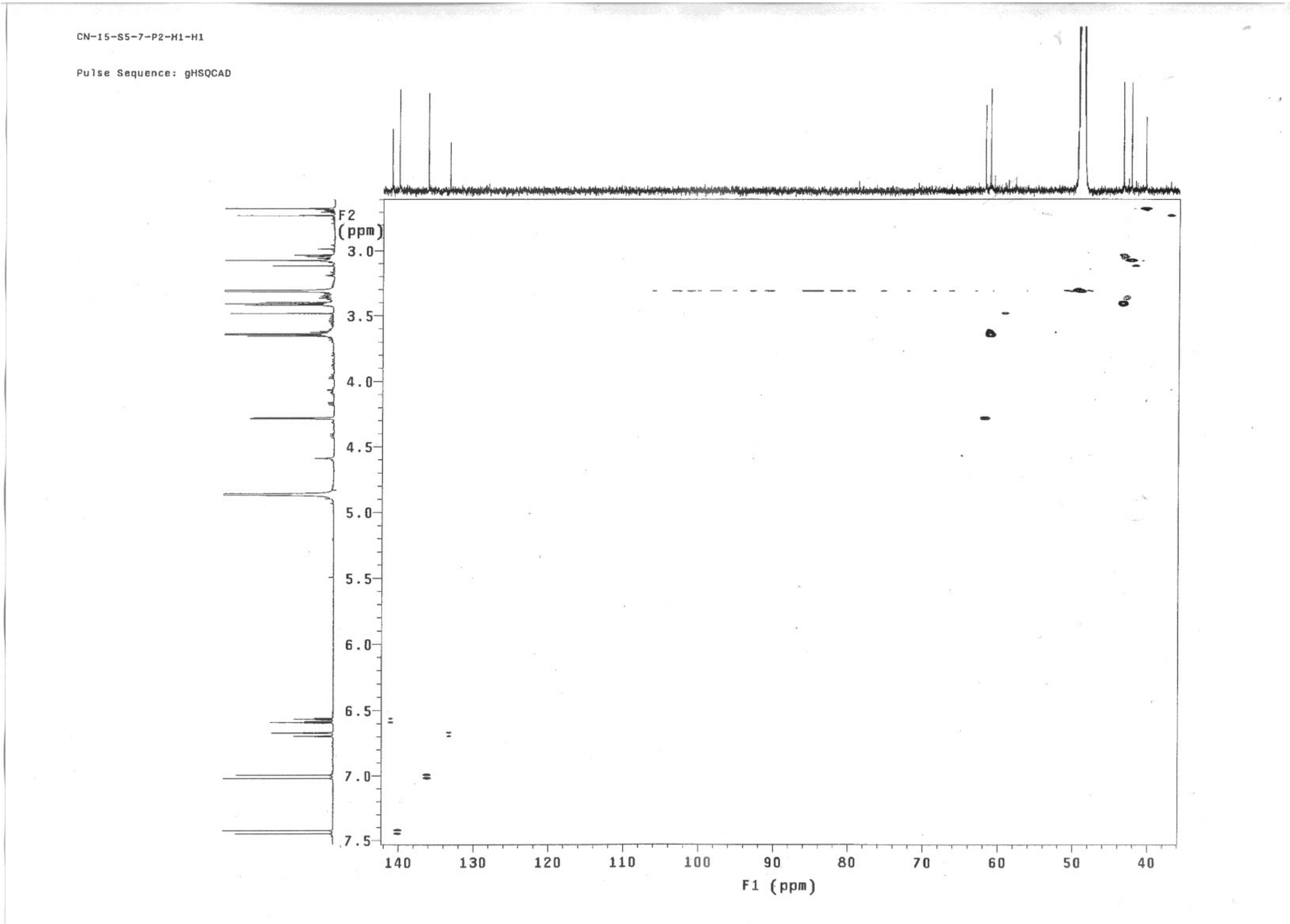

Figure S13. HMBC Spectrum of **2** in CD_3OD.

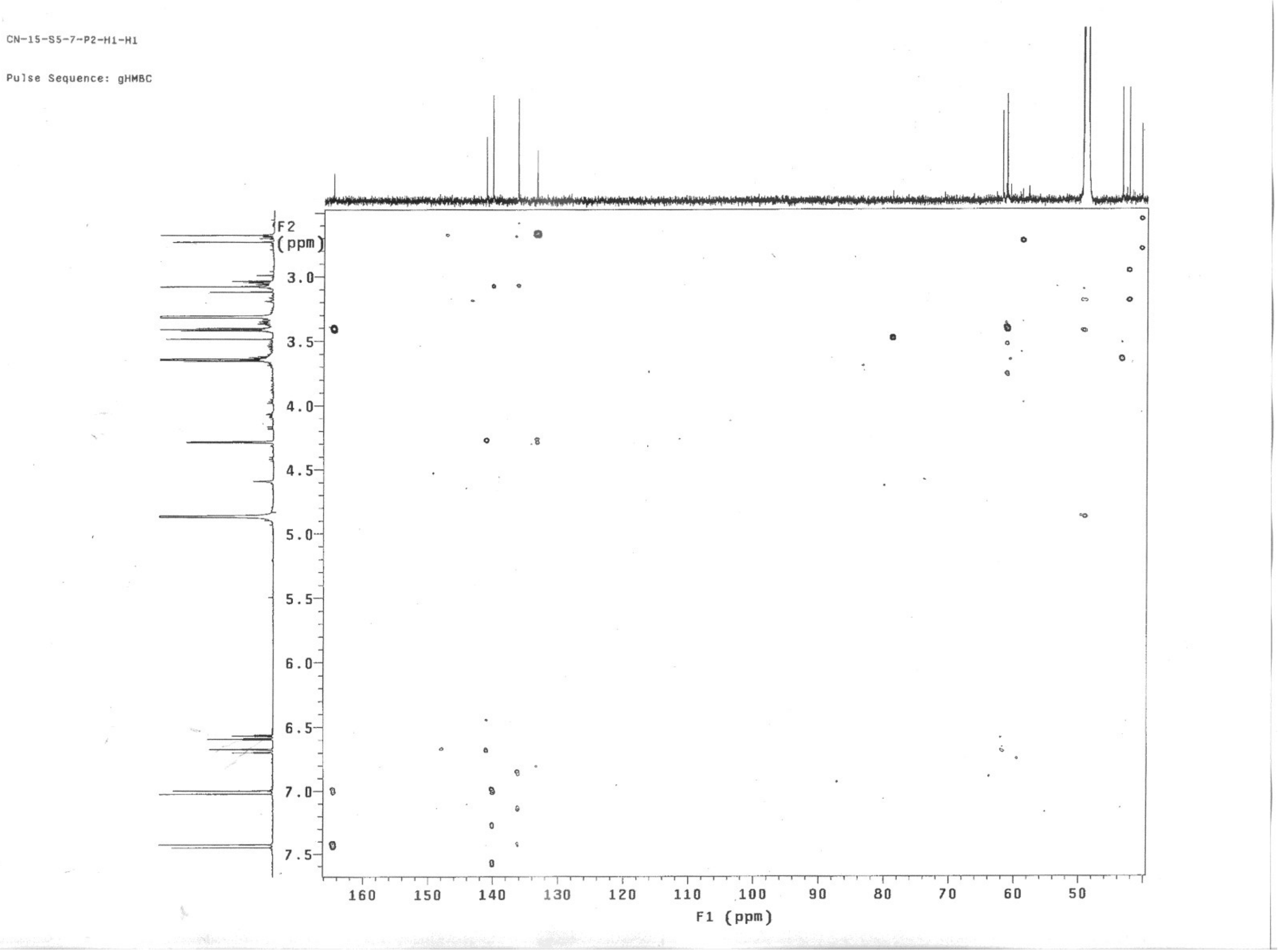

Figure S14. NOESY Spectrum of **2** in CD_3OD.

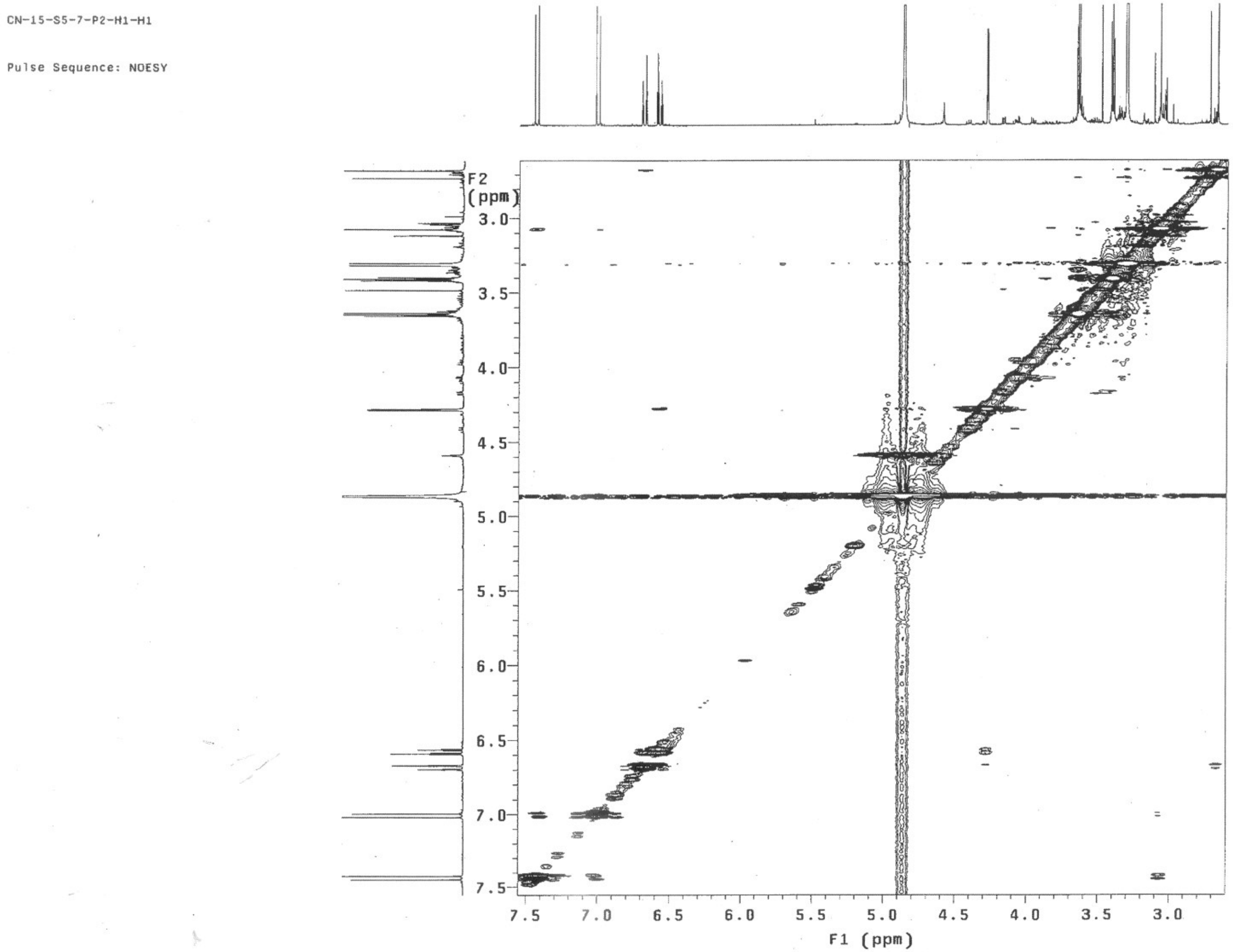

Figure S15. ^{1}H-NMR Spectrum of **3** in $CDCl_3$.

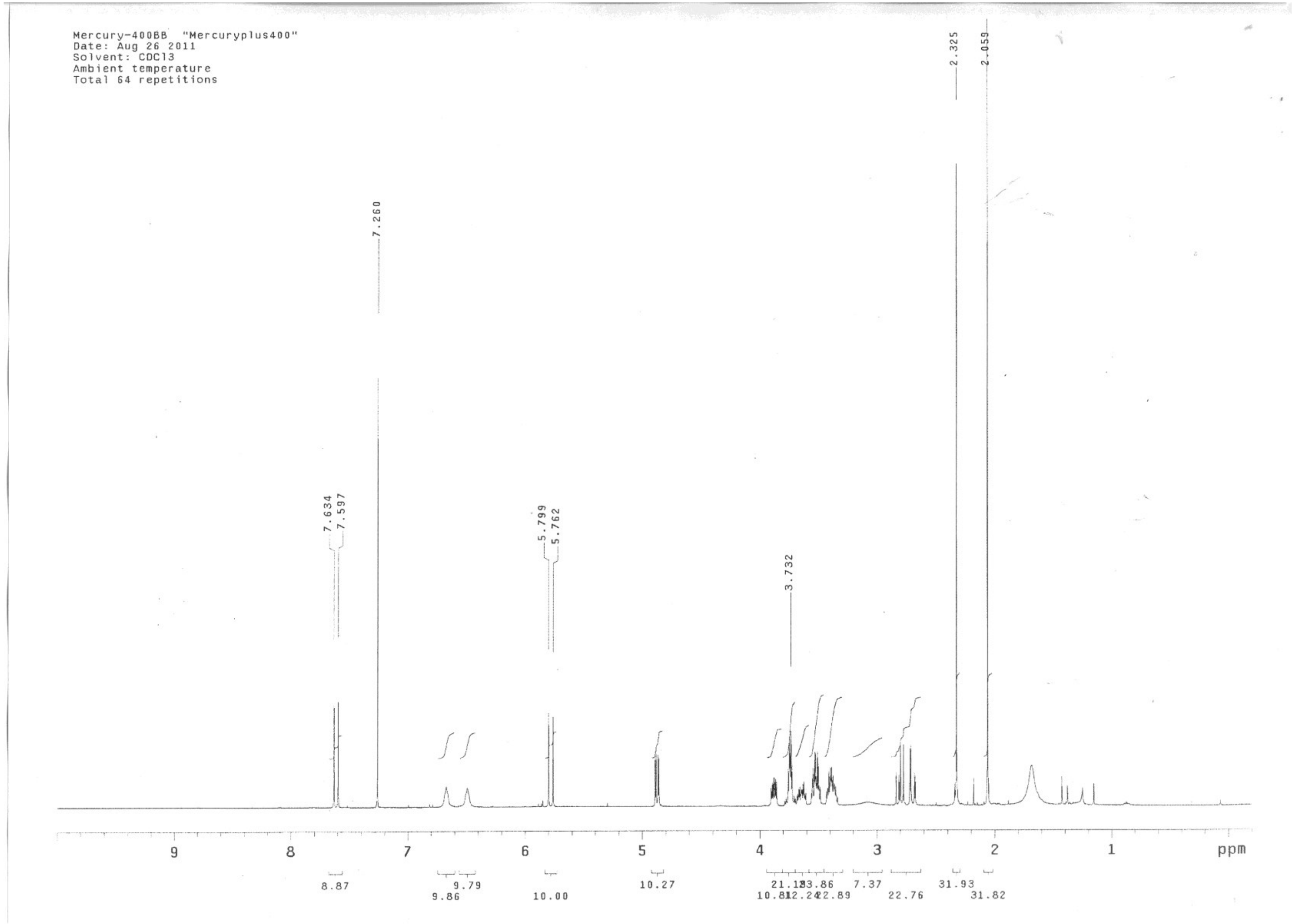

Figure S16. ^{13}C-NMR Spectrum of **3** in $CDCl_3$.

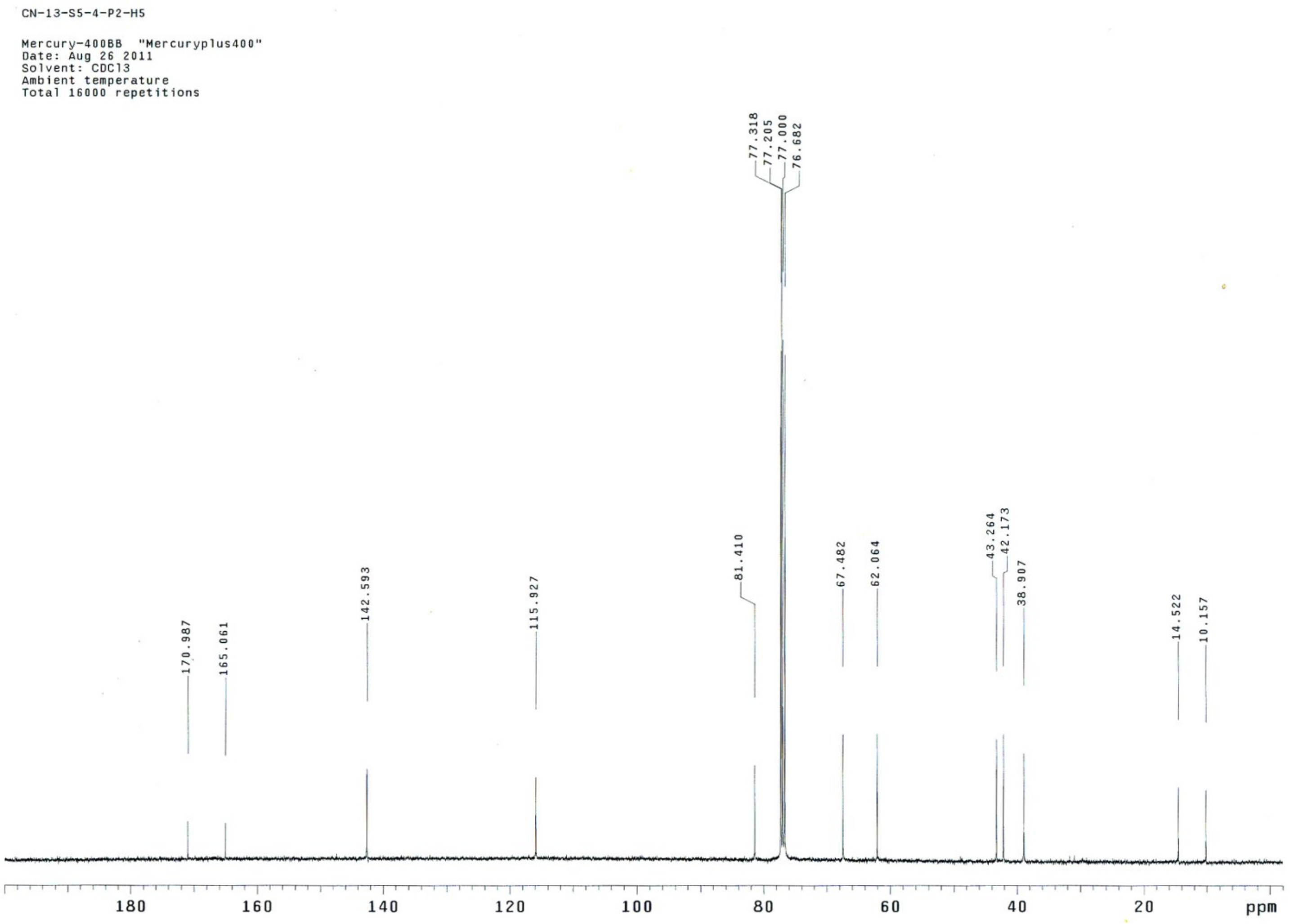

Figure S17. DEPT Spectrum of **3** in $CDCl_3$.

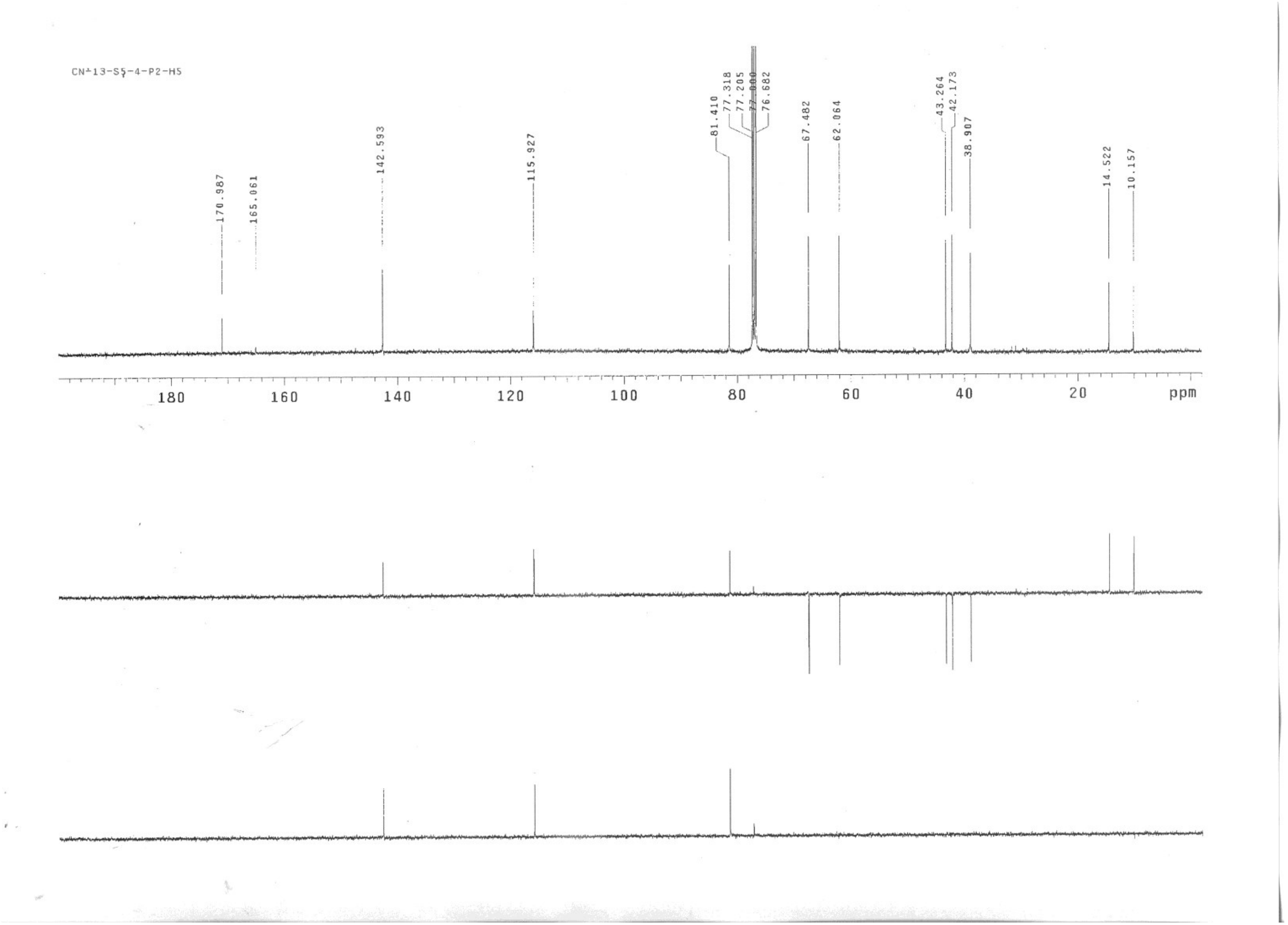

Figure S18. COSY Spectrum of **3** in $CDCl_3$.

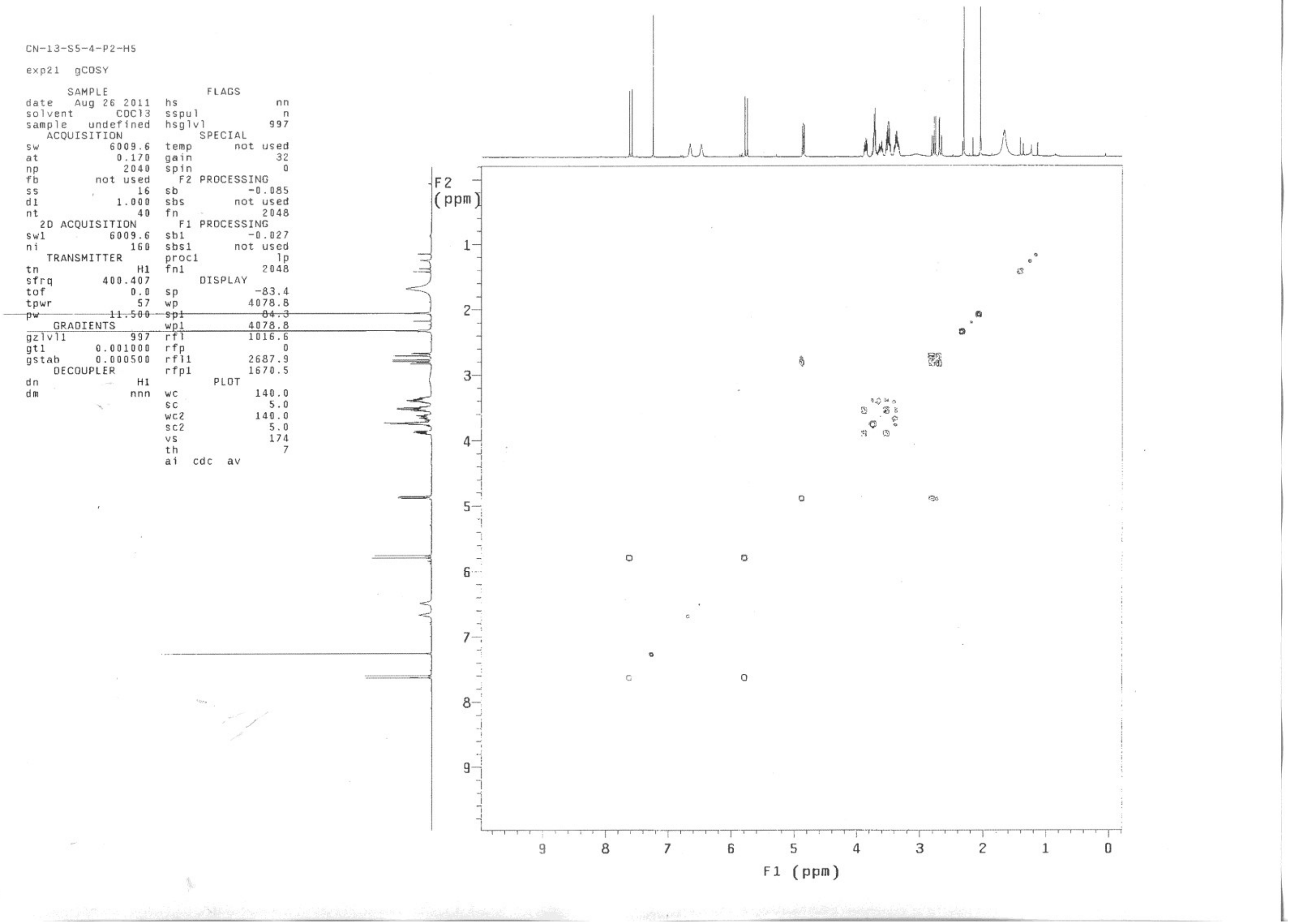

Figure S19. HMQC Spectrum of **3** in $CDCl_3$.

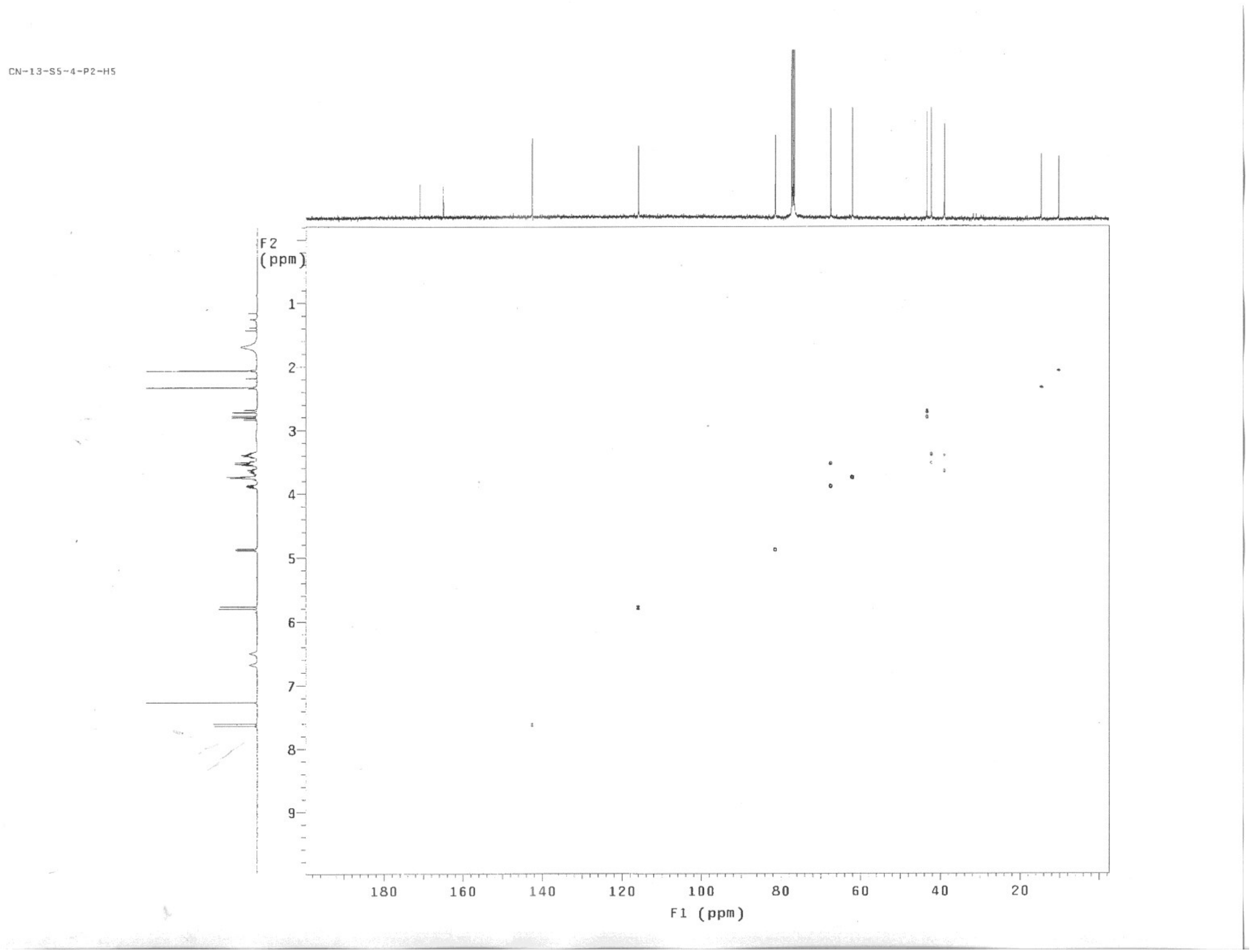

Figure S20. HMBC Spectrum of **3** in $CDCl_3$.

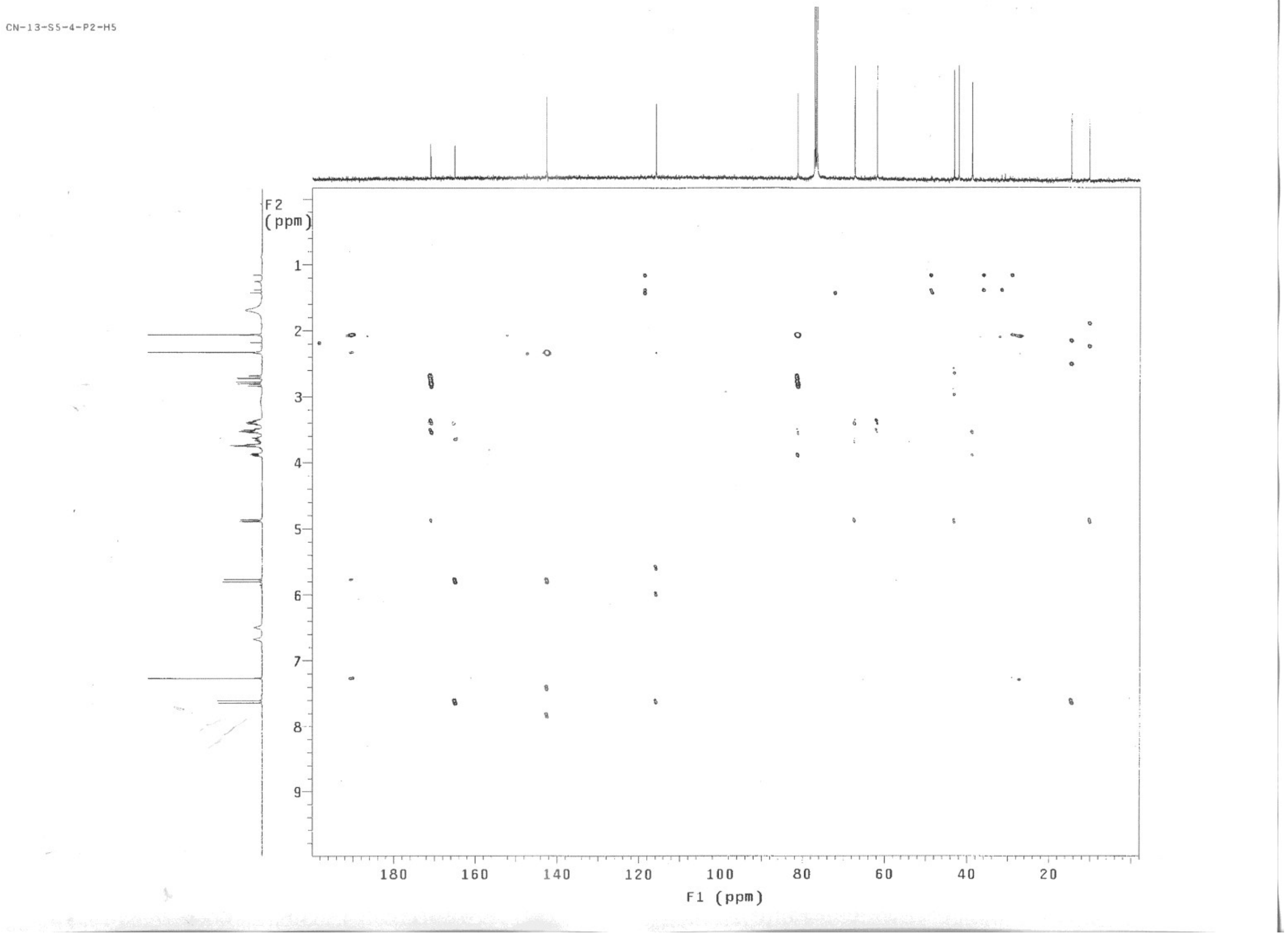

Figure S21. NOESY Spectrum of **3** in $CDCl_3$.

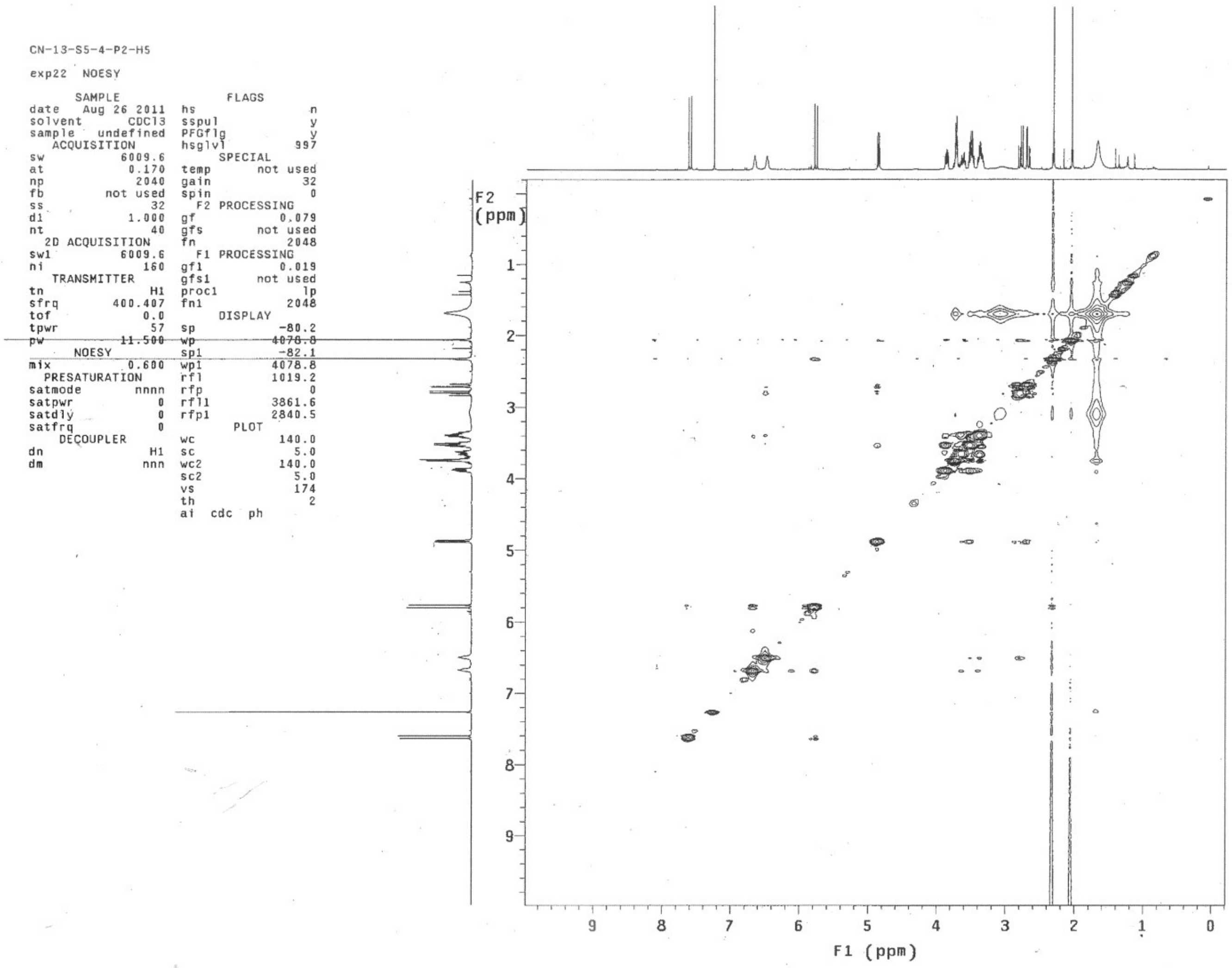

Figure S22. ^{1}H-NMR Spectrum of **4** in CDCl3.

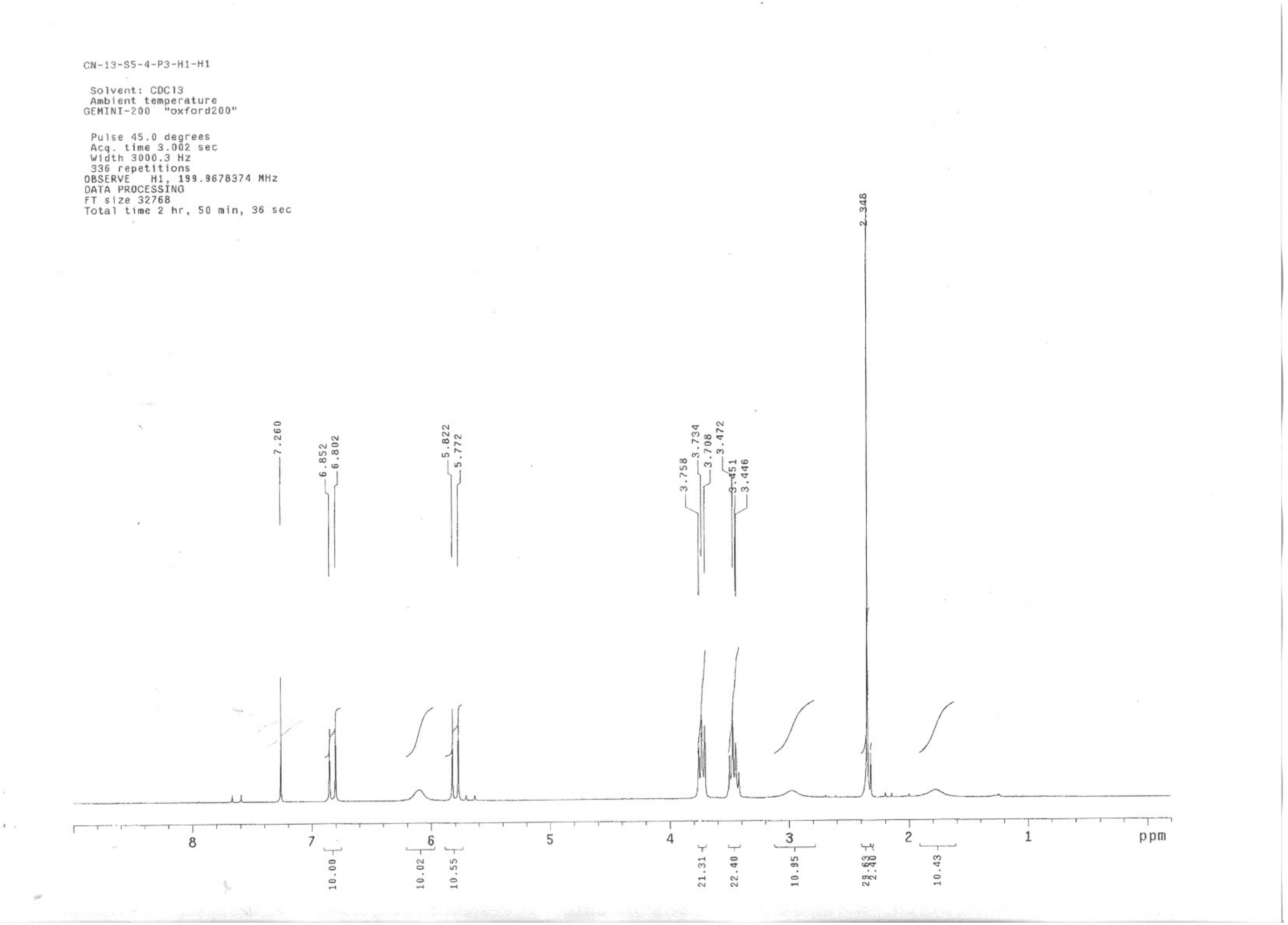

Figure S23. ^{13}C-NMR Spectrum of **4** in $CDCl_3$.

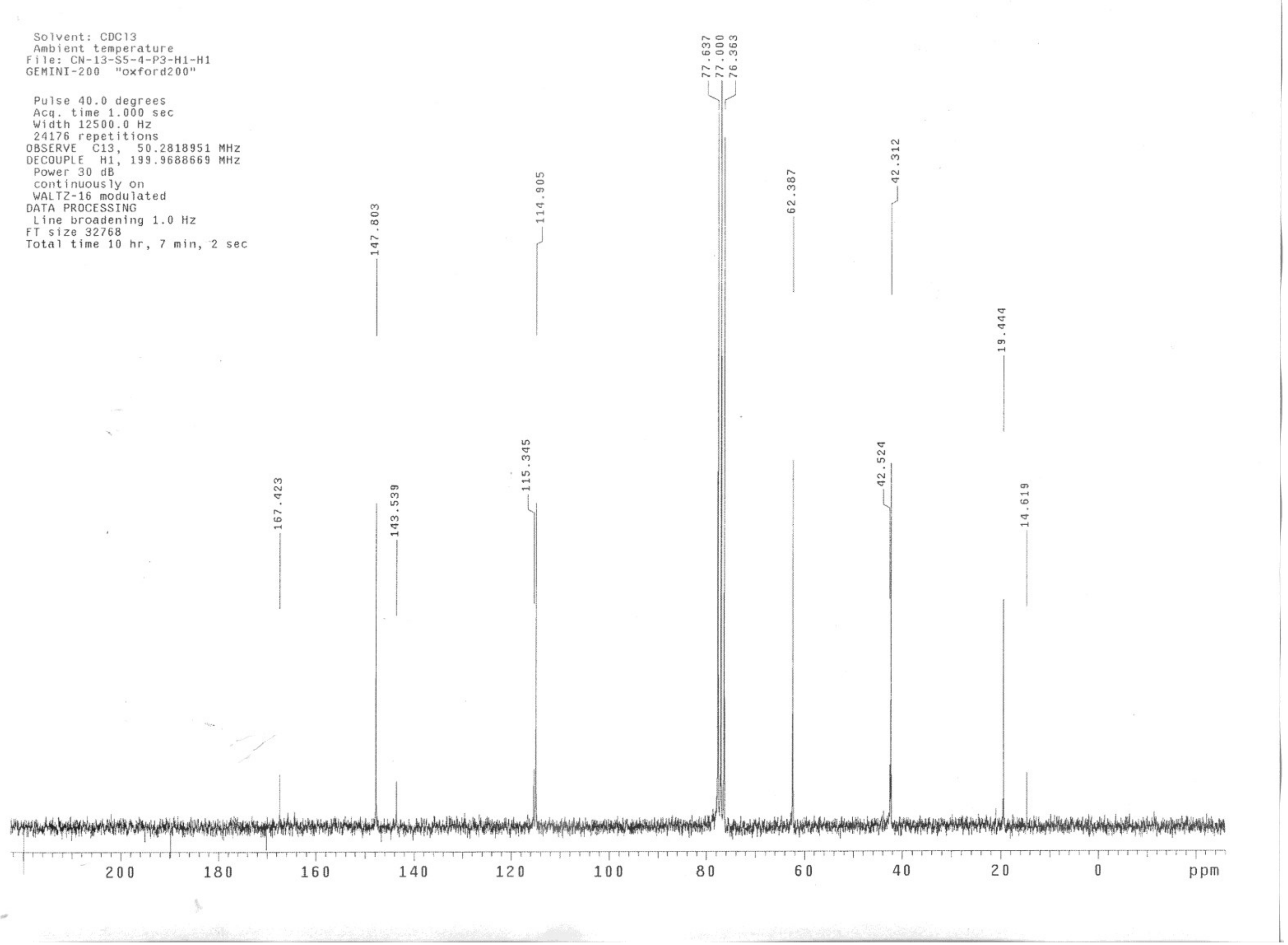

Table S1. Anti-inflammatory effects of ethanol extraction of *C. nutans* on superoxide anion generation and elastase release by human neutrophils in response to FMLP/CB.

Partition Layer	Superoxide Anion (Inh %)	Elastase Release (Inh %)
**EtOAc**	(8.58 ± 0.33) **	(60.53 ± 9.06) *
**80% EtOH**	(28.52 ± 2.55) **	(68.33 ± 5.49) **
***n*-hexane**	(13.40 ± 0.29) ***	(17.71 ± 7.54)

Percentage of inhibition (Inh %) at 10 μg/mL concentration. Results are presented as mean ± S.E.M. ($n = 2$). * $p < 0.05$, ** $p < 0.01$, *** $p < 0.001$ compared with the control value.

Figure S24. Anti-dengue virus 2 result of ethanol extraction of *C. nutans*.

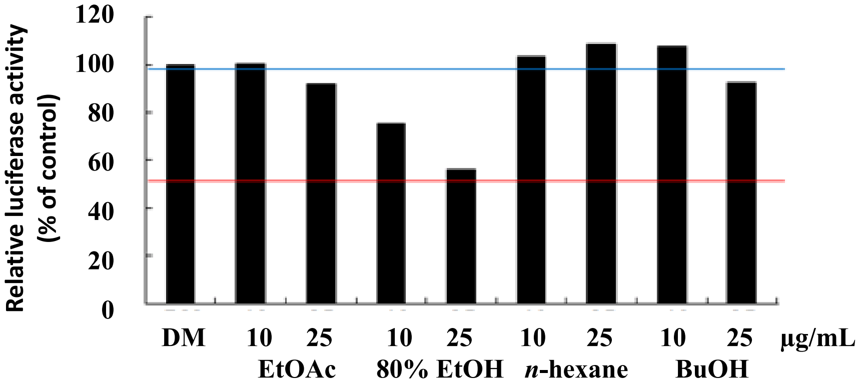

Figure S25. Immune-modulating result of 80% EtOH layer.

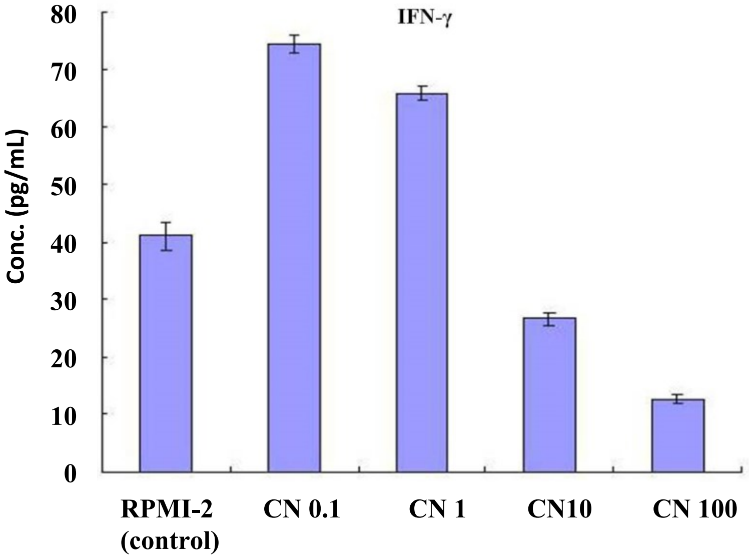

Supplement: Supplementary file 1 [file molecules-19-20382-s001.pdf]
